# CRISPR/Cas12a-RCA enables ultrasensitive detection of circulating free DNA for noninvasive diagnosis of *echinococcosis*

**DOI:** 10.1371/journal.pntd.0013069

**Published:** 2026-01-08

**Authors:** Jideng Ma, Yumei Zhang, Zian Li, Lanmin Liu, Jide A, Run Liang, Chunhua Cao, Jianwu Zhou, Peng Cheng, Yuqi Li, Zhiyuan Li, Li Ma, Lei Jiang, Xiangren A

**Affiliations:** 1 College of Clinical Medicine, Qinghai University, Xining, China; 2 Department of Clinical Laboratory, Qinghai Provincial People, s Hospital, Xining, China; 3 Center for Tibetan Medicine, Northwest Plateau Institute of Biology, Chinese Academy of Sciences, Xining, China; China Pharmaceutical University, CHINA

## Abstract

**Objective:**

To develop a novel non-invasive CRISPR/Cas12a-RCA assay for the primary screening of human *echinococcosis* via detection of circulating *Echinococcus* cell-free DNA (cfDNA) in peripheral blood.

**Methods:**

Plasma cfDNA from 20 AE patients was analyzed via high-throughput sequencing to identify conserved repetitive *Echinococcus* fragments.A one-pot RCA system coupled with CRISPR/Cas12a was optimized for *Echinococcus*-cfDNA detection. The limit of detection (LOD) was determined using serially diluted synthetic standards, while specificity was validated through mismatch probes and cross-reactivity testing. Clinical validation included 50 AE cases, 22 cystic echinococcosis (CE) cases, 43 non-*Echinococcus* hepatic disease (HD) cases, and 53 healthy controls (CON).

**Results:**

A conserved repetitive 28S rDNA fragment (pan-*Echinococcus*-28S) was identified as a biomarker. The CRISPR/Cas12a-RCA assay achieved amplification within 30 minutes at 37 °C, with a linear range of 1 aM to 100 pM and an LOD of 1.41 aM. Visual detection limits were 10 aM (UV light) and 1 aM (blue light). The assay demonstrated high sensitivity (87.5%) and specificity (96.9%, AUC = 0.933) in distinguishing *Echinococcus* infection (AE/CE) from HD and CON.

**Conclusion:**

The optimized one-pot CRISPR/Cas12a-RCA system enables rapid and ultrasensitive detection of pan-*Echinococcus* cfDNA, providing a cost-effective and highly accurate solution for the primary screening of echinococcosis.

## Author summary

*Echinococcosis* is a common parasitic disease in pastoral areas, mainly in areas of relative economic backwardness. There is no genetic test that can be used clinically. To improve this situation, we have developed a new test that identifies conserved genes for this disease by detecting a specific type of DNA in the blood. Our technique utilises DNA amplification and a precision cutting tool called CRISPR/Cas12a to quickly find tiny traces of parasite DNA. This method doesn’t require complex instrumentation or special laboratory conditions; it can be operated at room temperature and produces results in less than 30 minutes. We have tested it with blood samples from patients, and the results show that this method is not only fast and low-cost, but the accuracy of the results is also OK. This method provides a new option for the diagnosis and bedside testing of echinococcosis, helping doctors detect diseases more conveniently and thus avoid deterioration. Our research demonstrates how modern science can help solve real-world health problems by making medical testing more convenient and efficient.

## Introduction

Alveolar echinococcosis (AE), a chronic parasitic zoonosis caused by the larval stage of *Echinococcus multilocularis* (*Em*), carries a significant global disease burden of 666,434 disability-adjusted life years annually [1,2]. Without treatment, it has a mortality rate exceeding 90% within a decade [3,4]. Accurate diagnosis is crucial for reducing mortality; however, existing diagnostic methods have notable limitations [5,6]. Imaging techniques are ineffective for detecting lesions smaller than 2 cm in diameter and are unsuitable for patient screening [7]. Current antigen detection methods fail to identify specific antibodies in approximately 10–40% of patients with surgically confirmed parasitic infections [8,9]. Furthermore, as PCR assays require invasive sampling of tissue, cyst fluid, or cells—such as percutaneous biopsy or fine-needle aspiration—they carry a risk of allergic reactions due to accidental exposure to protoscolices or cyst fluid.[10]. Of particular concern is that the majority of AE patients reside in remote, pastoral regions where medical resources are limited [5]. By the time they reach healthcare facilities, their condition has typically advanced to later stages, resulting in the loss of the optimal treatment window [11,12].

Recent advances in cell-free DNA (cfDNA) as a non-invasive biomarker in oncology and prenatal screening have sparked interest in its application for AE diagnosis [13]. Parasite-derived cfDNA, released during larval proliferation and necrosis, has been detected in host blood and correlates strongly with lesion progression [14]. High-throughput sequencing (NGS) enables ultrasensitive detection of cfDNA; for instance, Fan et al. (2021) validated the utility of cfDNA in diagnosis and therapeutic monitoring [15], and it correlates with lesion size and stage [16]. NGS methods remain costly and technically demanding, while conventional PCR has a low sensitivity (positivity rate of 20–25 percent) [17]. Consequently, a significant gap exists between the proven diagnostic potential of cfDNA and the availability of a simple, rapid detection method that can fulfill point-of-care testing requirements.

In recent years, isothermal amplification technologies have emerged as promising tools for the molecular diagnosis of echinococcosis [18]. Studies [19] by Wassermann et and Salant demonstrated the practicality of Loop-mediated isothermal amplification (LAMP) for species identification and co-detection in field settings [20], while Ni highlighted its cost-effectiveness for canine surveillance [21]. Researchers have optimized a recombinase-aided amplification assay targeting the *12S rRNA* gene; however, most existing protocols were validated on animal or environmental samples, and their performance in human clinical specimens remains largely unexplored [22]. However, existing technologies still face significant challenges. LAMP and (Recombinase Polymerase Amplification) RPA rely on precise temperature control (65°C for LAMP and 37–42°C for RPA), which can be problematic in high-altitude areas with significant temperature fluctuations, potentially deactivating reagents [18,23,24]. Additionally, fluorescence-based detection requires specialized equipment such as thermostats or ultraviolet (UV) lights, which limits its applicability in resource-limited settings [25]. Multi-target assays also pose challenges, such as primer cross-amplification, which increases the risk of false positives [26]. Fluorescence detection, although effective, relies on specialized instrumentation and complex operational procedures, making it less suitable for point-of-care testing (POCT) scenarios [27].

CRISPR/Cas12a-RCA (CRISPR/Cas12a-integrated rolling circle amplification) is an exponential isothermal amplification system developed by modifying the conventional RCA with CRISPR/Cas12a integration.Its ambient-temperature reaction eliminates the need for temperature-controlled equipment [25,28]. Its high sequence specificity minimizes the risk of primer cross-contamination through precise pairing of circular templates [25]. Compared to conventional isothermal amplification methods, CRISPR/Cas12a-RCA has several key advantages [28,29]. It works well between 37–42°C, fitting high-altitude areas with temperature changes [28,30]. Its optimized design cuts nonspecific amplification under 0.1%, solving primer cross-reactivity in LAMP/RPA [30]. Additionally, amplified products are visible on lateral flow strips, eliminating the need for centrifugation or electrophoresis, which aligns with POCT requirements [31]. At present, clinical validation of this technology for *Echinococcus* cfDNA detection has not yet been conducted.

This study aimed to develop a CRISPR/Cas12a-RCA system for the detection of *Echinococcus* cell-free DNA (cfDNA), with the goal of providing a new, accessible POCT option for resource-limited setting.

## Materials and methods

### CfDNA sequencing and specific fragment screening

#### Ethics statement.

Plasma samples were prospectively collected from January 2023 to September 2024 at Qinghai Provincial People’s Hospital and Golo state People’s Hospital under ethical approval (License No. 2023–069) and a human genetic resource collection permit (License No. 2023CJ0212). Written informed consent was obtained from all participants.

#### Patients.

Experimental Cohort: AE Patients: Plasma was collected from 24 confirmed AE cases prior to surgical or pharmacological intervention. The diagnosis was validated by imaging (ultrasound and CT) and histopathology. Controls: Plasma was obtained from 5 patients with non-echinococcal parasitic infections.Validation Cohort: AE Group: 50 patients with varying AE severity.Disease Controls: Cystic echinococcosis (CE): 22 cases.Other Hepatic Conditions: 43 cases, including hepatocellular carcinoma (n = 12), hepatic cysts (n = 17), liver abscesses (n = 8), hepatic hemangiomas (n = 4), and metastatic liver cancer (n = 2).Healthy Controls: 53 age-matched individuals with no hepatic abnormalities. Plasma aliquots were stored at −80°C until analysis. Demographic, diagnostic, and therapeutic parameters were recorded, including Initial infection timeline, Lesion diameter (imaging-confirmed), and Treatment history (surgery, albendazole regimen).The current study was conducted in Xining City, Qinghai Province, China, which is located on the Qinghai-Tibet Plateau at an altitude of 2261 meters.

#### Peripheral blood cfDNA extraction and library preparation.

Peripheral blood samples were processed immediately after collection. Whole blood was centrifuged at 1,600 × g for 10 min (4°C) to separate plasma from cellular components. The supernatant was transferred to a 1.5 mL microcentrifuge tube and subjected to a secondary centrifugation step (16,000 × g, 10 min, 4°C) to pellet residual cellular debris [33].cfDNA was extracted from 4 mL of clarified plasma using two parallel methods to ensure yield and purity: Centrifugal Column-Based Isolation: QIAamp MinElute cfDNA Kit (55114, QIAGEN, Germany). Magnetic Bead-Based Purification: QIAseq cfDNA All-in-One Kit (TP709, QIAGEN, Germany). Extracted cfDNA was quantified using a Qubit 2.0 Fluorometer (Thermo Fisher Scientific, USA) with the Qubit dsDNA HS Assay Kit, optimized for low-concentration nucleic acids (<1 ng/μL). For library construction, 30 ng of plasma cfDNA was fragmented and ligated with adapters using the QIAseq FX DNA Library Kit (108310, QIAGEN, Germany) following the manufacturer’s protocol. Final libraries were stored at −80°C until sequencing was performed.

#### Sequencing of cfDNA.

Sample quality was evaluated through DNA integrity assessment on 1% agarose gel and quantitative concentration measurement using Qubit. Twenty samples from the AE group and two from the control group met the quality criteria. High-throughput sequencing was performed using an Illumina HiSeq X-series sequencer with optional PE150 sequencing mode. Generated data were removed from splice sequences and unqualified data using the tool trim-galore (version 0.4.4) (select paired-end to remove reads <20 bp and Phred scores below 20). Finally, FastUniq (version 1.1) was used to remove duplicate sequences [32].

#### Characteristics of sequenced clinical samples.

Among 24 AE patients and 5 parasite controls screened, 20 AE cases and 2 control samples qualified for library construction. The sequencing cohort comprised 3 early-stage, 8 intermediate-stage, and 9 late-stage AE cases, along with two control specimens: one *Clonorchis sinensis* infection and one *Taenia solium* infection.AE patients (age range: 9–59 years; mean±SD:37.05 ± 11.44) included 19 Tibetans and 1 Han Chinese individual, with comparable gender distribution (11 females vs. 9 males). Hepatic involvement was predominantly right-lobe localized (n = 15). Metastatic disease was observed in 40% of cases (8/20), with pulmonary involvement in 87.5% (7/8) of metastatic cases, including left-lung (n = 4), bilateral lung (n = 2), and right-lung (n = 1) metastases. One case exhibited concurrent bilateral pulmonary and cerebral metastases. Extrahepatic invasion was documented in three specimens (abdominal cavity, pancreas, and diaphragm). The cohort consisted primarily of treatment-naïve patients (17/20), with three recurrent cases. Detailed demographic and clinical characteristics are summarized in Table 1.

**Table 1:** Information on clinical samples for sequencing.

	Patient Information							cfDNA sequencing			
ID	Age	Gender	Nationality	Lesion Location	S/M	I/R	EAb	Raw reads (PE)b	Clean reads	*Echinococcus* Reads	*Echinococcus* RPMc
**AE2**	39	Female	Tibetan	Bilateral liver	M	I	0.46	72549260	72480948	8207	113.2297552
**AE4**	27	Female	Tibetan	Right hemiliver	S	I	3.4	62380860	62315656	10458	167.8229946
**AE5**	33	Female	Tibetan	Bilateral liver, Bilateral lung; Abdomen	M	I	4.23	64291150	64228734	10884	169.4568665
**AE6**	59	Female	Tibetan	Bilateral liver	S	I	1.35	96217066	96130706	7077	73.61851686
**AE7**	34	Male	Tibetan	Right hemiliver; Bilateral lung;	S	R	2.53	65677646	65619324	9381	142.9609363
**AE8**	33	Female	Tibetan	Bilateral liver; Bilateral lung; Left brain	M	R	5.69	99971188	99878548	15492	155.1083822
**AE9**	43	Male	Han	Bilateral liver	M	I	2.45	75553148	75482292	8252	109.3236543
**AE10**	35	Male	Tibetan	Left hemiliver; Left lung	S	I	3.16	110672044	110568636	15904	143.8382581
**AE11**	42	Female	Tibetan	Bilateral liver; Left lung	M	I	4.75	101078644	100985574	15778	156.2401378
**AE12**	37	Female	Tibetan	Right hemiliver	S	I	7.42	88172810	88092794	14070	159.7179447
**AE13**	52	Female	Tibetan	Right hemiliver	S	I	2.32	90177052	90093922	8845	98.17532419
**AE14**	45	Male	Tibetan	Right hemiliver	S	I	1.12	48410548	48369658	3251	67.21155647
**AE15**	9	Male	Tibetan	Right hemiliver; Diaphragm	S	I	10.7	94550168	94460996	17953	190.0572804
**AE16**	40	Male	Tibetan	Bilateral liver	M	I	0.96	74437066	74367814	7065	95.00077547
**AE17**	43	Female	Tibetan	Right hemiliver; Left lung; Pelvis;	S	R	4.11	101385148	101290762	13442	132.7070676
**AE18**	29	Female	Tibetan	Left hemiliver	S	I	1.85	78353168	78280078	8017	102.4143078
**AE19**	41	Male	Tibetan	Right hemiliver	S	I	1.45	101677718	101585234	10884	107.1415556
**AE21**	18	Male	Tibetan	Right hemiliver, Right lung	M	I	3.06	87922650	87842834	12026	136.9035976
**AE22**	51	Male	Tibetan	Left hemiliver, Left lung	M	I	6.63	74375432	74312906	14791	199.0367595
**AE24**	31	Female	Tibetan	Right hemiliver	S	I	2.7	77313694	77240846	11834	153.2090935
**Con1**	24	Male	Tibetan	NA	NA	NA	0.12	136433282	135208272	3257	24.08876285
**Con 5**	28	Female	Tibetan	NA	NA	NA	0.23	36500926	36130850	2118	58.62026495

Notes: S: Single lesion; M: Multiple lesions; I: Initial infection; R: Recurrence; EAb: *Echinococcus* antibody; PE: Paired-end Reads; RPM: Read-Pairs Per Million

#### Analytical informatics data analysis.

Bioinformatics Analysis Workflow:(1)Raw Data Processing: Sequencing raw image files was converted into FASTQ-formatted reads (RawData) through base calling. These files contained both nucleotide sequences and corresponding Phred quality scores. (2)Quality Control: Adapter sequences and low-quality bases (Phred score <20) were removed using Trimmomatic, yielding filtered Clean Reads. Post-QC data volumes ranged between 5.37–20.10 GB.(3)Reference Genome Alignment: Clean reads were aligned to the *Em* reference genome (NCBI accession: GCA_000469725.3) using Bowtie2 [33]. Alignment files were sorted and converted to BAM format using SAMtools, with mapping statistics subsequently calculated. (4)Chromatin Immunoprecipitation DNA Sequencing: Genomic read distribution was analyzed using MACS2 for peak identification. A sliding window approach was implemented to detect regions of read enrichment. Potential peaks were statistically validated through Poisson distribution modeling, with false discovery rate (FDR)-adjusted q-values calculated. Peak length distributions were analyzed to determine cfDNA fragmentation patterns. Peaks were annotated based on genomic features (exons, coding regions, intergenic regions) using BEDTools and the reference genome’s annotation file—differential Peak Analysis. Cross-group comparisons of peak read counts were performed using DiffBind to identify differentially enriched regions. Functional Enrichment Significantly enriched GO terms and KEGG pathways were identified with topGO (v2.36.0) and clusterProfiler (v3.12.0). Terms/pathways with p-values <0.05 after the Benjamini-Hochberg correction were retained. Detailed methods for Chromatin Immunoprecipitation DNA Sequencing are provided in S1 File.

#### Repetitive cfDNA fragment screening.

Sequences potentially associated with *Echinococcus* species were prioritized by filtering out fragments that showed high homology to the human reference genome. Candidate sequences were first validated through BLASTn alignment against the *Em* database (NCBI: txid6211), retaining only high-confidence matches (E-value < 1e-5). Recurrent cfDNA fragments observed in patients with cystic echinococcosis were systematically compared. To ensure taxonomic specificity, all fragments alignable to the human genome were excluded using BLASTn against the NCBI human database.

#### Quantitative PCR.

QPCR was conducted using an MA-6000 system (Yarui Biotech, China) with TB Green Premix Ex Taq II FAST (RR830B; Takara Bio Inc., Japan). β-Actin served as the endogenous reference gene. Each 20 μL reaction contained five μL master mix, 10 μL cfDNA template, and one μL primer mixture (5 μM each). Thermal cycling parameters included: initial denaturation at 95°C for 3 min, followed by 41 cycles of 95°C for 5 s (denaturation), 55°C for 5 s (annealing), and 72°C for 20 s (extension). Relative cfDNA concentrations were calculated using the 2 − ΔΔCt method. All samples and negative controls were analyzed in triplicate to ensure reproducibility.

### CRISPR/Cas12a-RCA method for cfDNA measurement

#### Principle of CRISPR/Cas12a-RCA method.

The method for extracting cfDNA is consistent with the processing method for sequencing samples. Genomic DNA was removed from the samples using the RNEasy Plus Mini Kit (Qiagen, Cat. No: 74134).The process begins with hybridization between a locked-loop probe and *E. multilocularis* cfDNA (*Em*-cfDNA) targets, enhanced through rapid denaturation and annealing during sample preparation. The 5’-phosphorylated locked-loop probe contains two functional modules: a linker region complementary to *Em*-cfDNA for target bridging and a detection region recognized by the Cas12a-crRNA ribonucleoprotein (RNP) complex. Following hybridization, T4 DNA ligase circularizes the probe-target complex to form a template for RCA. Phi29 DNA polymerase then extends the circular template, generating long tandem repeats complementary to the locked-loop sequence. Preloaded Cas12a RNP binds to these repeats, activating two functions: (1)trans-cleavage activity that non-specifically degrades fluorophore-quencher-labeled ssDNA reporters, producing real-time fluorescent signals; and (2)cis-cleavage activity that fragments RCA products into short DNA primers. These primers initiate secondary RCA cycles, converting linear amplification into exponential signal generation.

Workflow Overview:(1)A split-connector locked-loop probe is designed to bind target cfDNA and activate Cas12a-crRNA recognition. (2)Extracted cfDNA is hybridized with the probe and ligated into circular templates. (3)RCA amplification and Cas12a-mediated cleavage occur in a one-pot reaction containing phi29 polymerase, Cas12a RNP, and fluorescent reporters. (4)Fluorescence is monitored in real time using a standard PCR instrument at 37°C. The coupling of Cas12a’s dual cleavage activities with RCA enables ultrasensitive detection, overcoming the limitations of conventional PCR. Collateral ssDNA reporter cleavage further amplifies signals, enhancing sensitivity for low-abundance cfDNA targets. The detailed steps of the One-Pot CRISPR/Cas12a-RCA Assay Protocol are provided in S2 File, while the Optimised step-by-step RCA-CRISPR Protocol is outlined in S3 File.

#### Primer and probe design and synthesis.

Primers and probes for CRISPR/Cas12-RCA detection were designed in DNAMAN software using the target cfDNA sequence (ACGAGATCCCTACTGTCCCTATCTACTATCTAGC) and synthesized by Shanghai Sangong Biotechnology Co., Ltd.(1)RCA Probe Design: A stem-loop probe forRCA was optimized to target AE-associated cfDNA repeats:5′GACAGTAGGGATCTCGTGTGGA[cattatatgatcgagagagttgcccgcatgtgtttc]GCTAGATAGTAGATAGG-3′;(2)crRNA Design:crRNA-M1 was designed based on the target DNA sequence and PAM motif positioning to ensure specificity for *Echinococcus* derived nucleic acids:5′-TAATTTCTACTCTTGTAGATTGCTCTAGGGGATGACAGGGGATAGATGATAGATCG-3′;(3)Fluorescent Reporters: Trans-cleavage reporter: FAM-TTATTT-BHQ1 (universal signal probe) Cis-cleavage reporter: FAM-TGCTCTAGGGATGACAGGGATAGATGATAGATCG-BHQ1.

#### Materials for isothermal amplification.

The MA-6000 qPCR system (Yarui Biotech, China) was operated at 37°C for 50 minutes (single cycle). The enzymes and reagents used included dNTPs (25 mM), Phi29 DNA polymerase (0.1 U/μL), SplintR ligase (25 U/μL), T4 DNA ligase (1000 U/μL), and Cas12a protein (1 μM) (New England Biolabs, USA). Additional materials were sourced from Thermo Fisher Scientific (USA) and included the following biochemicals: ATP, DTT, and BSA; detection reagents: SYBR Green II dye and SYBR Gold nucleic acid stain; and electrophoresis supplies: agarose and 10 × TBE buffer.

#### Optimization of RCA reaction conditions.

The necessity of six key components—T4 DNA ligase, Cas12a nuclease, 5p probe, phi29 DNA polymerase, crRNA, and a fluorescently labeled probe—was evaluated. Control experiments were designed by systematically omitting each component individually. Its impact on reaction outcomes was assessed to confirm its role in the CRISPR/Cas12a nucleic acid detection system. Enzyme activity is temperature-dependent. Experiments were conducted at 16°C, 20°C, 25°C, 30°C, 35°C, 37°C, and 42°C to determine the optimal reaction temperature for Cas12a nuclease activity and detection efficiency.Reaction duration directly influences the efficiency of enzyme-substrate binding and cleavage. Time intervals of 10, 30, 60, 90, and 120 minutes were tested to identify the optimal reaction time for maximal signal output. This systematic approach ensures precise optimization of ligation, amplification, and detection phases, enabling robust and reproducible CRISPR/Cas12a-RCA assay performance.

#### Optimization of RCA reaction system.

(1) Enzyme Concentration Screening: Phi29 DNA polymerase: Volumes of 0.05 ~ 0.2 μL (0.1 U/μL) were tested, with other conditions held constant.T4 DNA ligase: Volumes of 0.3 ~ 0.6 μL were evaluated under fixed parameters.Cas12a-crRNA complex: Concentrations of 0.5 ~ 3 μL (premixed at a 1:2 ratio) were assessed. (2)Padlock Probe Optimization: Seven padlock probes were screened using 1 pM *Em*-cfDNA.Probe concentrations (50 ~ 1,000 nM) were tested for optimal binding efficiency (S1 Table for probe details). Buffer Compatibility Testing Laboratory-modified buffers: Versions T5 ~ T9 were compared.Kit-supplied buffers: Cas12a, T4, and phi29 buffers were evaluated. The Phi29 buffer volume, ranging from 1 to 3 μL, was tested with other parameters held constant. (3)Reagent Titration: Fluorescent probe: Volumes of 1 ~ 3 μL were optimized.BSA: 1 ~ 4 μL was tested for nonspecific binding reduction.ATP:0.5 ~ 1.5 μL was added to ATP-free reactions.DTT: 20 ~ 50 mM concentrations (2 μL) were evaluated.dNTPs: 0.5 ~ 3 μL (25 mM stock) were tested for amplification efficiency. All experiments were conducted using a single-variable adjustment approach to isolate the effects of each parameter.

#### Sensitivity analysis.

A concentration gradient of *Em*-cfDNA fragment standards was prepared through serial dilution, spanning from 100 pM to 1 aM (100 pM, 10 pM, 1 pM, 100 fM, 10 fM, 1 fM, 100 aM, 10 aM, 8 aM, 6 aM, 5 aM, 4 aM, 2 aM, 1 aM). Each concentration was analyzed using the RCA method, with three biological replicates included for every dilution level.

#### Specificity analysis.

(1)Non-target DNA Testing: Six mismatched *Em*-cfDNAs (100 nM, 100 pM, 100 fM, and 100 aM) were tested using the CRISPR/Cas12a system to evaluate cross-reactivity. (2)Clinical Sample Validation: Four parasitic infection samples *(Taenia solium, Clonorchis sinensisscaris, Plasmodium vivax, Ascaris lumbricoides*) confirmed by clinical diagnosis were obtained from Qinghai Provincial People’s Hospital. (3)Synthetic miRNA Screening: Seven synthetic miRNAs (*emu-miR-1-3p*, *emu-miR-10-5p*, *emu-miR-7-5p*, *emu-miR-9-5p*, *emu-let-7-3p*, *emu-miR-novel 1–3*), synthesized by Shanghai Biotechnology Co., were diluted to 1 pM and analyzed.

#### Agarose gel electrophoresis.

A 3% agarose gel prepared in TBE buffer was electrophoresed at 110 V for 1 hour. The gel was stained with SYBR Gold dye in TBE buffer for 30 minutes. Imaging was performed under 254 nm UV light using an Odyssey Infrared Imaging System (LI-COR).

### Statistical methods

Data are presented as mean ± SEM. Receiver operating characteristic (ROC) analyses were conducted using GraphPad Prism v10.1 (GraphPad Inc.). Statistical analyses were performed using GraphPad Prism and SPSS 26 (SPSS Inc., Chicago, IL, USA). Spearman’s rank correlation method was applied for correlation analyses. A two-tailed significance threshold (α = 0.05) was adopted, with *P* < 0.05 indicating statistical significance.

## Results

### CfDNA sequencing and specific fragment screening

#### Analysis of Sequencing Results.

**Fig 1:**
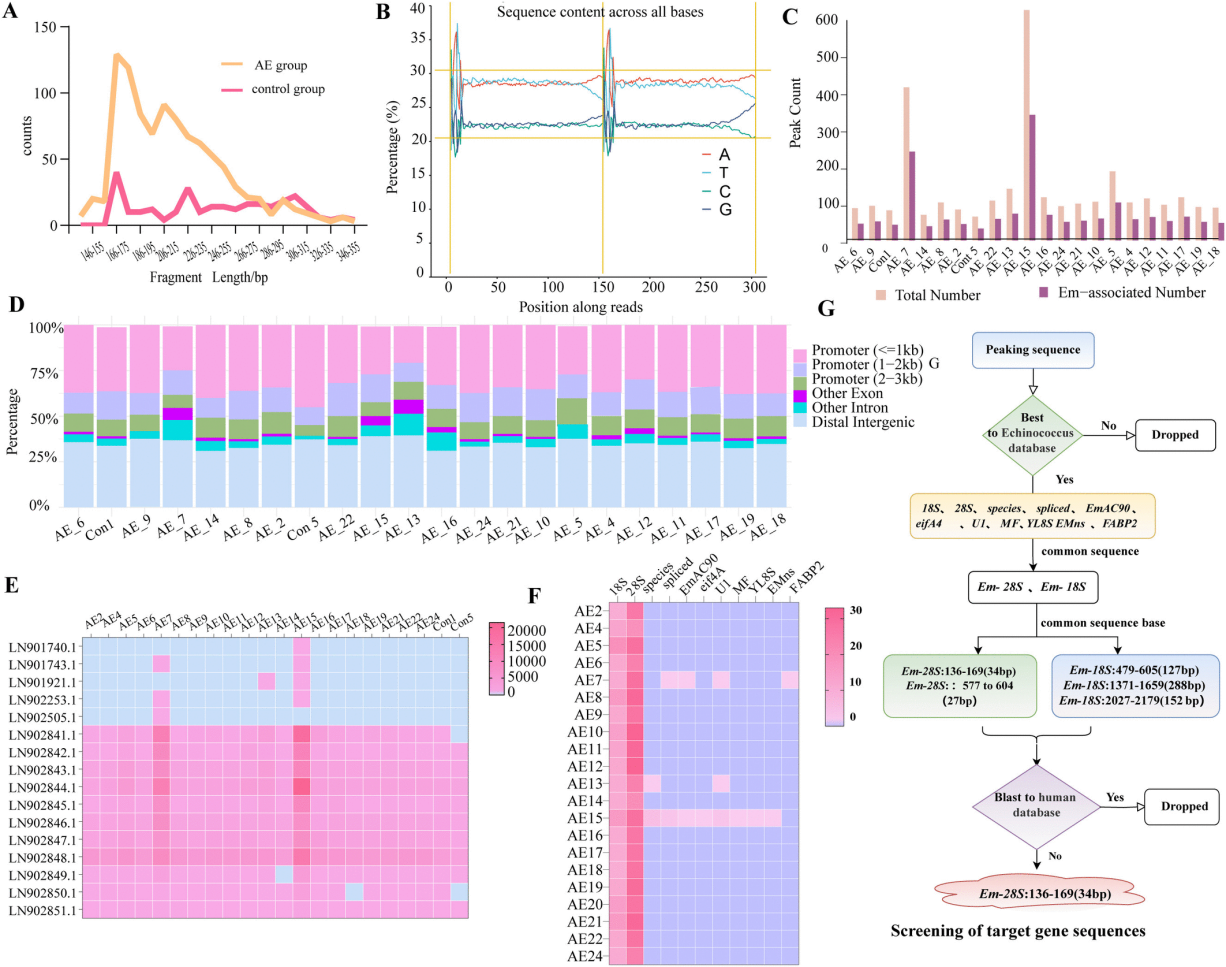
Analysis of sequencing results. **(A):**Fragment length distribution in the AE group versus controls, with fragment size (bp) on the horizontal axis and read counts on the vertical axis;**(B):**Nucleotide composition across read positions, showing the percentage of A/T/C/G bases (vertical axis) at each nucleotide position (horizontal axis);**(C):**Peak distribution of candidate sequences, with sequence types on the horizontal axis and corresponding peak counts on the vertical axis;**(D):**Genomic region composition across samples, displaying the percentage of sequences in promoter regions (<1kb, 1-2kb, 2-3kb), exons, introns, and distal intergenic regions (color-coded bars) for each sample;**(E):**Sample-specific sequence distribution, illustrating the number of unique sequences (vertical axis) per sample (horizontal axis);**(F):**Quantitative analysis of candidate sequences, showing sequence counts (vertical axis) across samples (horizontal axis);**(G):**Target gene screening workflow, including peak sequence selection, alignment with *echinococcus* genomes, and human genome filtering.

A reference database for *Echinococcus* spp. was constructed using plasma cfDNA sequencing data (S1 Fig). Clean reads (5.37 ~ 20.10 GB) were obtained through quality filtering and mapped to the *Em* genome, yielding 2,118 ~ 17,953 aligned reads (0.01 ~ 0.02% of total clean reads). This refinement process enhanced data accuracy and improved the quality of the reference database. Significant differences in fragment length distributions were observed between AE and control groups (Fig 1A). *Em*-cfDNA predominantly spanned 100 ~ 200 bp, while control samples showed enrichment in shorter fragments (50 ~ 150 bp). Base composition analysis revealed similar A/T/C/G profiles across groups, although distinct positional base biases in AE samples suggested epigenetic modifications (Fig 1B). Fragment length-specific peaks were most prominent in metastatic samples AE7 and AE15 (Fig 1C). Sequence-type distribution analysis demonstrated preferential enrichment of *Em*-cfDNA in promoter-proximal regions (≤1 kb: > 50%) compared to distal regions (1–3 kb: < 20% combined) (Fig 1D). Exonic sequences constituted 21.5% of AE cfDNA versus <5% in controls, indicating infection-associated transcriptional activation—exhaustive flowchart of the microarray sequencing (chip seq) analysis of *Em*-cfDNA(S2 Fig).AE samples exhibited elevated expression of genes on loci LN902841.1 ~ LN902851.1 (Fig 1E), which are implicated in cell cycle regulation and immune responses. Control samples exhibited minimal variation in expression across these loci. Comparative analysis against conserved sequences (*18S/28S rDNA*, *EmAC90*,*eif4A*, *UI*, *M*, *YL8S, EMns*, *FABP2*) revealed high sequence similarity (>85%) in AE-derived fragments (Fig 1F). Gene-specific annotations are provided in S1 Table. A multi-step bioinformatic pipeline was implemented (Fig 1G): (1) Primary screening against the *Echinococcus* genome database; (2) Filtering of sequences homologous to conserved elements (*18S/28S rDNA, EmAC90,eif4A*); (3) Final exclusion of human-homologous sequences. Collectively, AE-associated cfDNA exhibited distinct fragmentomic features, sequence composition biases, and transcriptional regulatory signatures compared to controls, providing critical insights into host-parasite interactions. In the AE group, the *Echinococcus* Reads (PE) and RPMc values were significantly higher than those in the control group, with values of 11181.1 ± 3763.2 and 133.7 ± 36.71 for AE, compared to 2688 ± 805.4 and 41.35 ± 24.42 for CON, respectively. The t-test results indicated statistically significant differences between the groups (P values of 0.0054 and 0.0026, respectively).The results of the analysis of cfDNA sequencing subgroup information are shown in Table 2.

**Table 2:** Subgroup analysis of cfDNA sequencing results.

		n	*Echinococcus* Reads (PE)b	*t/F*	*P*	*Echinococcus* RPMc	*t/F*	*P*
**AE stage**	early	3	7699.8 ± 2161.2	14.4	0.0002	95.8 ± 16.8	25.26	<0.0001
	middle	8	12120.7 ± 1823.0			160.3 ± 7.32		
	advancd	9	13961.2 ± 2750.1			158.5 ± 23.3		
**Metastasis**	M	8	13462.3 ± 2466.3	2.502	0.0222	154.5 ± 21.5	2.299	0.0337
	S	12	9659.4 ± 3778.2			119.7 ± 38.8		
**Recurrence**	R	3	12771.7 ± 3110.2	0.7864	0.4419	143.6 ± 11.2	0.4982	0.6243
	I	17	10899.8 ± 3878.9			131.9 ± 39.5		

Notes:S: Single lesion; M: Multiple lesions; I: Initial infection; R: Recurrence; PE: Paired-end Reads; RPM: Read-Pairs Per Million

#### Screening and validation of specific cfDNA fragments.

Specific cfDNA fragments were screened and validated through systematic analysis of plasma cfDNA sequencing data from 20 AE patients and two controls. Eleven sequences showing high homology with the *Em* genome (taxid:6211) were identified by BLAST alignment, corresponding to ribosomal RNA (*28S/18S*), non-structural proteins (*EMns*), and functional gene (*FABP2*) regions. Conserved regions in 28S (*Em-28S*) and 18S (*Em-18S*) ribosomal genes were further characterized through multiple sequence alignment. Three segments were identified in *Em-18S* (127 bp, 288 bp, and 152 bp), while two regions were defined in *Em*-28S: 136–169 nt and 577–604 nt. The five cfDNA repeat sequence targets were validated using the Rolling Circle Amplification (RCA) method. For detailed information, please refer to S3 Fig. Detailed information on the *Em-28S* Gene Sequence Alignment Results of the AE2 sample is provided in S2 Table.

Following cross-validation against the human genome, *Em-28S*:136–169 was selected as the optimal marker (S3 Table). This fragment was detected in 85.0% (17/20) of AE patients but was absent in controls and a healthy cohort (n = 50) (*P* < 0.001). Conventional PCR proved ineffective for cfDNA detection in this study, highlighting the need for more sensitive detection methods.

### Construction and optimisation of one-pot CRISPR/Cas12-RCA

#### CRISPR/Cas12a-RCA Reaction System.

**Fig 2:**
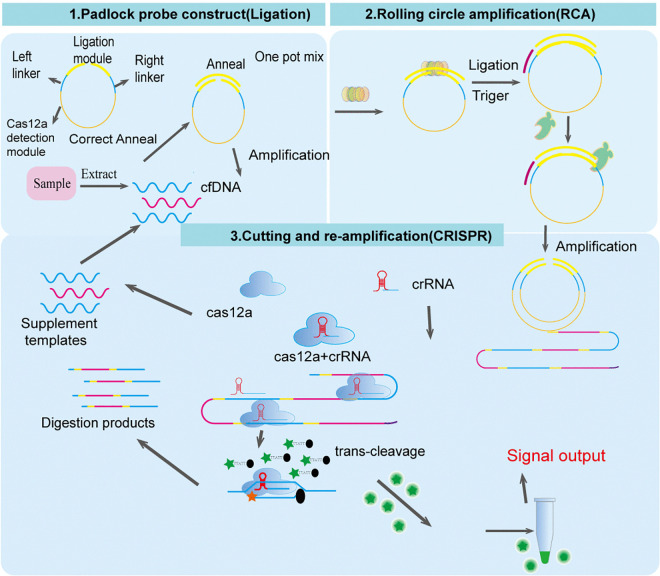
Schematic illustration of the one-pot *CRISPR/Cas12a-RCA* assay for cfDNA detection. The *CRISPR/Cas12a-RCA* detection system consists of two key components: (1) a circular DNA probe containing a target-specific hybridization region and a CRISPR/Cas12a activation sequence, and (2) a reaction mix containing Cas12a-crRNA ribonucleoprotein (RNP), fluorescent single-stranded DNA (ssDNA) reporters, and RCA enzymes.In the procedure, extracted cfDNA is hybridized with the circular probe. Following hybridization, the probe-DNA complex is added to the reaction system and incubated at 37°C in a real-time qPCR instrument. Target recognition initiates rolling circle amplification (RCA), generating repetitive ssDNA products. These products activate the Cas12a-crRNA complex through sequence complementarity, triggering two simultaneous enzymatic activities: (i) specific cleavage of RCA-amplified ssDNA to release secondary primers for exponential amplification, and (ii) non-specific cleavage of fluorescent reporters to generate measurable signals. Real-time fluorescence monitoring enables quantitative analysis of target cfDNA.

**Fig 3:**
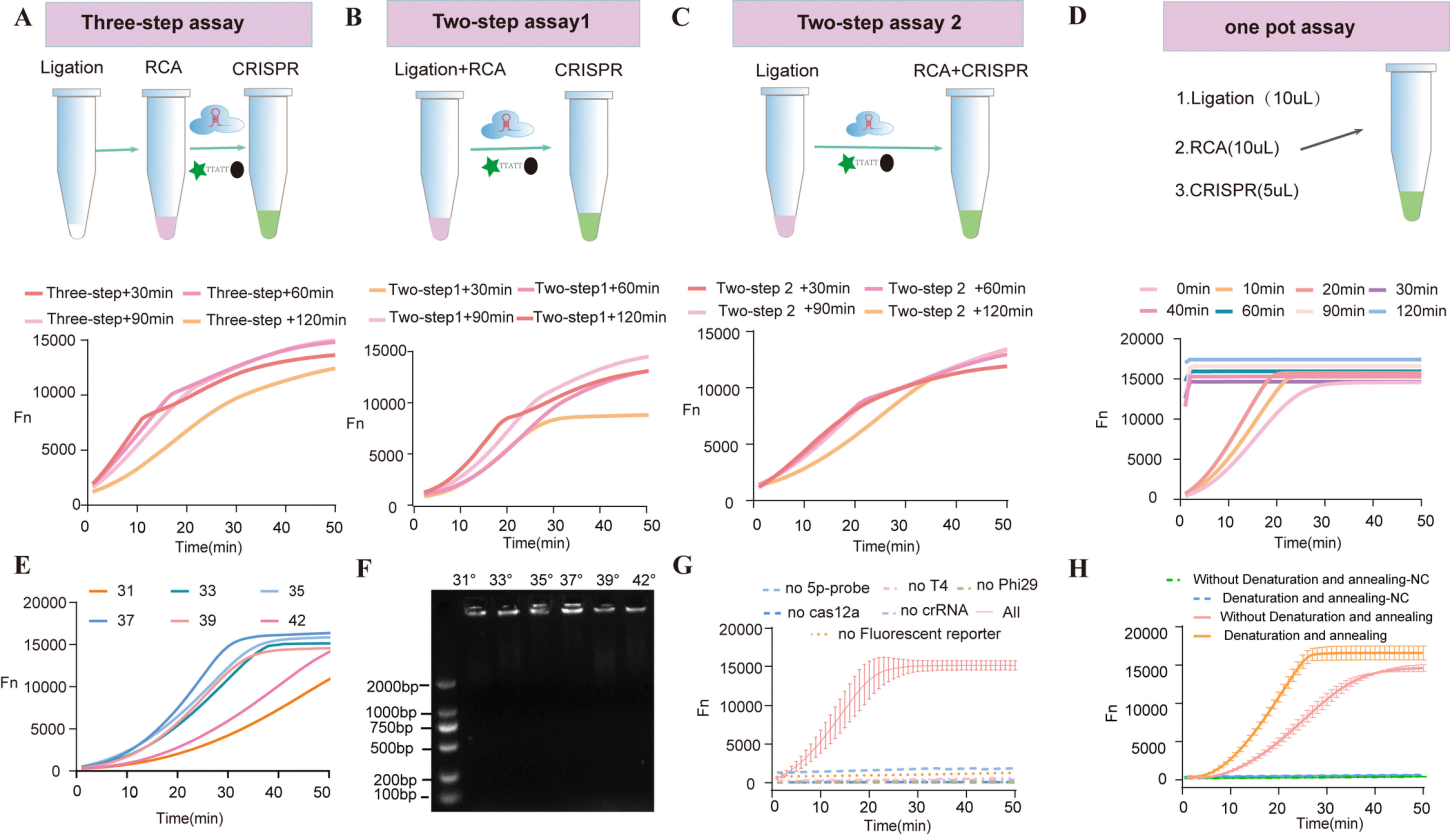
Optimization of the *CRISPR/Cas12a-RCA* reaction system. **(A):**Three-step workflow: Three critical reaction steps were time-optimized: (1) Ligation with T4 DNA ligase (37°C, 30-120 min), (2) Amplification with phi29 DNA polymerase (37°C, 30-120 min), and (3) Cas12a-mediated detection (directly loaded into the instrument). Fluorescence curves were generated with 100 fM EG-cfDNA;**(B):**Two-step method 1: Combined ligation-RCA reactions (variable incubation times) followed by Cas12a-crRNA addition for cleavage. **(C):**Two-step method 2: Ligation reaction (variable durations) prior to sequential RCA and Cas12a RNP addition for fluorescence detection;**(D):**One-pot assay: Simultaneous integration of ligation, amplification, and Cas12a detection in a single tube;**(E):**Temperature-dependent fluorescence kinetics, showing real-time signal acquisition across thermal conditions;**(F):**Agarose gel analysis of amplification products at different temperatures;**(G):**Component validation: Six essential reagents (ligase, polymerase, Cas12a, crRNA, dNTPs, probes) were systematically tested for functional necessity;**(H):**Annealing requirement validation post-ligation: Signal intensity (Y-axis: Fn) was monitored over time (X-axis: 1-min cycles), with negative control (NC) included.

The principle of the CRISPR/Cas12a-RCA approach is illustrated in Fig 2. The correlation between RCA efficiency and reaction time was evaluated using time-course experiments. As shown in Fig 3A, fluorescence signal intensity in the three-step system exhibited a strong positive correlation (*R²* = 0.98) with reaction duration (30 ~ 120 min). Signal intensity plateaued at 120 minutes, showing a 3.2-fold increase compared to the 30-minute group (*P* < 0.001), confirming that extending the reaction time enhanced the exponential amplification of the circular template.Only 18.6% of the total signal increase occurred within the first 30 minutes.

In the stepwise system (Fig 3B and 3C), the timing of Cas12a nuclease addition critically influenced sensitivity. Delayed Cas12a introduction after 120 minutes of RCA (two-step method) yielded a 2.7-fold higher fluorescence intensity than the 30-minute group, demonstrating that maximal signal output occurs when CRISPR cleavage follows sufficient RCA amplification. By contrast, the one-pot system (Fig 3D) exhibited superior kinetics, with a fluorescence increase rate 1.8 times faster than the stepwise method, and plateaued at 60 minutes. This improvement was driven by three factors: Multi-enzyme synergy, reducing intermediate product loss (42.3% higher retention verified by qPCR). Temperature gradient experiments (Fig 3E and 3F) identified 37°C as optimal, at which phi29 polymerase achieved peak processivity, outperforming conditions at 35°C and 40°C. Key component exclusion tests (Fig 3G) revealed 98.2%, 95.7%, and 99.3% signal reductions upon omitting ligase, circular template, or Mg² ⁺ , respectively, confirming each component’s essential role. Annealing pretreatment (Fig 3H) lowered the detection limit from 0.1 pM to 5 fM by resolving template secondary structures and enhancing crRNA targeting efficiency. The optimized one-pot CRISPR/Cas12a-RCA system achieved a 100-fold increase in sensitivity over traditional stepwise methods, establishing a robust framework for ultrasensitive nucleic acid detection. As shown in S4 Fig, in-gel recycling was performed. However, the T4-PHI29-CAS12A reaction was not successfully initiated when Multicopies were used as templates, probably because the repetitive sequences in the Multicopies degradation products might have interfered with the enzyme recognition and activity. When Singlecopies was used as a template, a clear signal was observed, especially at higher cycle numbers, suggesting that the Singlecopies degradation product was able to initiate the T4-PHI29-CAS12A reaction efficiently.

#### Optimization of components.

**Fig 4:**
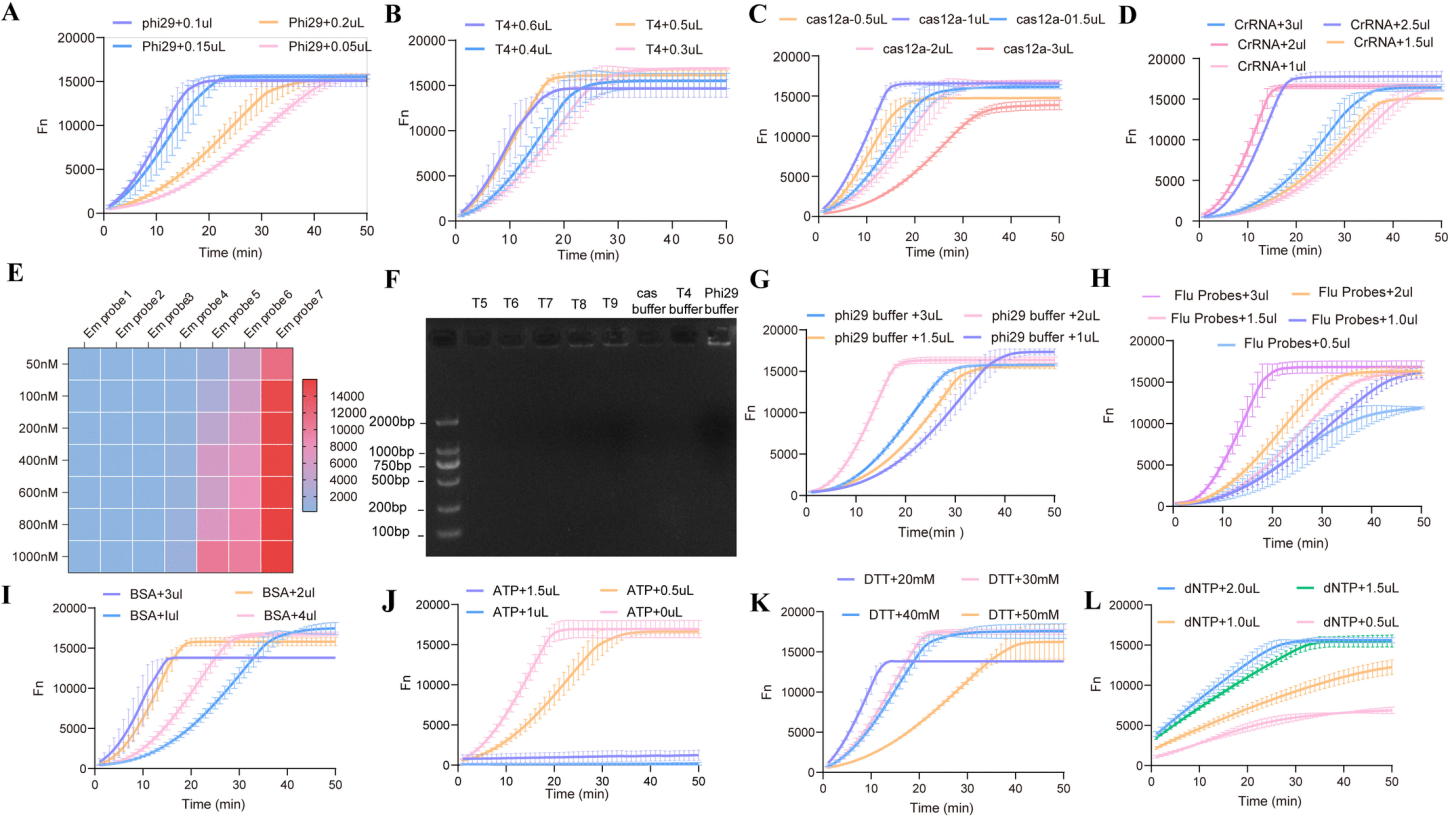
Optimization of one-pot *CRISPR/Cas12a-RCA* for EM-cfDNA detection. **(A):**Phi29 polymerase dosage optimization. Optimal fluorescence signals (Fn) were achieved with 0.1 μL of 10 U/μL phi29 polymerase;**(B):**T4 DNA ligase dosage optimization. Peak signal intensity was observed at 0.5 μL of 10 U/μL T4 DNA ligase;**(C):**Cas12a enzyme dosage optimization. Maximum fluorescence occurred with 1 μL of 10 U/μL Cas12a;**(D):**crRNA concentration screening. **(E):**Padlock probe concentration effects. Fluorescence intensity was evaluated with 1 pM EM-cfDNA and 50–1000 nM probes;**(F):**Buffer system comparison. Amplification efficiency was analyzed by gel electrophoresis using 1 pM EM-cfDNA and 100 nM probes, testing commercial buffers (Cas12a/T4/phi29) versus lab-modified buffers (T5-T9);**(G-L):**Component optimization. Effects of phi29 buffer, fluorescent probes, BSA, ATP, DTT, and dNTP concentrations were systematically evaluated under fixed conditions.All experiments included triplicate replicates with negative controls (NC). Fluorescence intensity (Fn) was monitored over reaction time (1-min cycles). Raw FAM channel fluorescence curves are displayed for condition optimization.

The RCA reaction system was optimized through systematic adjustment of enzyme components. As shown in Fig 4A, the concentration of phi29 DNA polymerase critically influenced reaction kinetics. A low concentration (0.05 μL) caused delayed signal amplification, while 0.1 μL achieved optimal efficiency, yielding a maximum relative fluorescence unit (RFUmax) of 12,450 ± 320 through enhanced DNA synthesis. Excess enzyme (>0.15 μL) induced premature reaction termination, likely due to enzyme aggregation. T4 DNA ligase optimization identified a critical threshold for primer-template ligation (Fig 4B). A ligation efficiency of 92.7% was achieved at 0.5 μL (qPCR analysis). In contrast, concentrations greater than 1.0 μL increased nonspecific ligation by 38%. The Cas12a/crRNA system exhibited a strict stoichiometric dependence (Fig 4C and 4D), with maximum amplification efficiency achieved at a 1:1 molar ratio (0.1 μM Cas12a to 1 μL of 10 μM crRNA). Higher concentrations caused a 2.1-fold increase in off-target DNA cleavage. Emprobe screening identified Probe 7 as optimal, demonstrating a 3.8-fold fluorescence enhancement over alternatives (Fig 4E). CRISPR/Cas12a-RCA padlock 5p probe Sequences are in S4 Table—probe concentrations greater than 100 nM produced a plateau effect consistent with saturation kinetics. Buffer optimization revealed that the commercial phi29 buffer outperformed in-house formulations (T5-T9), increasing the double-stranded DNA yield by 23% (Fig 4F), with a volume of two μL being determined as the optimal volume (Fig 4G). The FAM reporter exhibited concentration-dependent self-quenching (Fig 4H). Peak signal intensity (18,340 RFU) was observed at one μL (100 nM), while 200 nM resulted in a 22% signal attenuation. The addition of 2 μL BSA improved reaction stability (Fig 4I). Unexpectedly, exogenous ATP inhibited amplification (Fig 4J), with two mM ATP causing complete suppression. The DTT concentration showed a narrow activity window (Fig 4K). dNTP titration identified 0.5 μL as optimal for balancing the extension rate and fidelity (Fig 4L). Laboratory indicators for *echinococcus* and controls are in S5 Table.

#### Assay system specificity and sensitivity validation.

**Fig 5:**
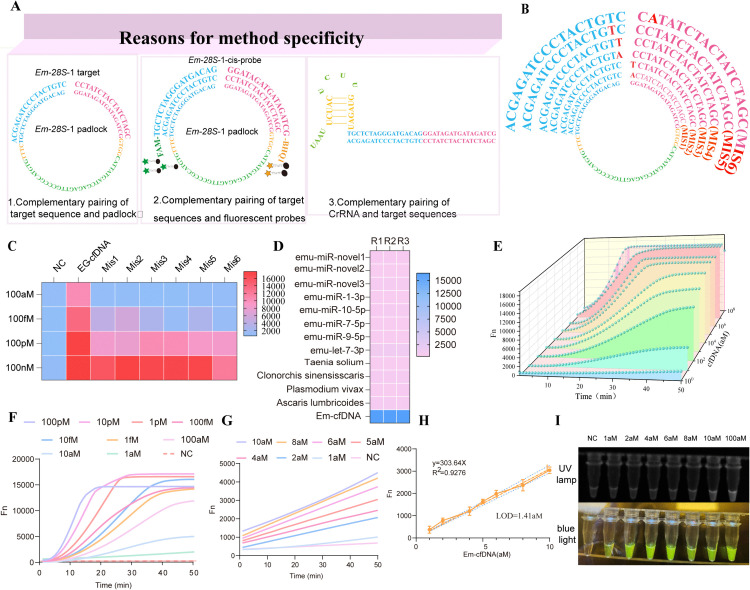
Sensitivity and specificity of the *CRISPR/Cas12a-RCA* assay for cfDNA detection. **(A):**Three mechanisms contributing to assay specificity;**(B):**Specificity validation through six-base mismatches. Mismatch positions are highlighted in the sequence schematic;**(C):**Fluorescence heatmap at cycle 50, demonstrating differential signals between matched and mismatched targets; **(D):**Specificity assessment. No cross-reactivity was observed with cfDNA from four parasitic pathogens or eight non-target miRNAs;**(E):**Sensitivity gradient analysis. Synthetic Eg-cfDNA standards were tested from 1 aM to 100 pM, including no-template controls (NC);**(F):**Real-time amplification curves of Eg-cfDNA detection across concentrations;**(G-H):**Standard curves for low-concentration targets (1-10 aM). Titration curves in G and H exhibit time-dependent performance, with signal-to-noise ratios influenced by reaction module positioning in padlock probes;**(I):**Optimized protocol validation.Representative fluorescence curves (left) and endpoint signals under UV/blue light (right) confirm concentration-dependent detection.

The assay’s specificity is attributed to a three-stage molecular recognition mechanism **(Fig 5A)**: Locked probe-target complementary binding. Thermodynamic control enables precise cyclization via 20 nt terminal sequences that are complementary to the target. Fluorescent probe amplification product hybridization: A FAM-labeled probe forms a 25 bp duplex with RCA product repeats, activating Cas12a trans-cleavage activity.crRNA-target sequence alignment: A 34 nt crRNA achieves target recognition through its 5′ seed sequence. The CRISPR/Cas12a system demonstrated efficient discrimination of single-base mismatches. In validation experiments, six mismatch probes (Fig 5B) produced detectable signals at target concentrations of≥100 fM. However, the exact-match probe exhibited significantly higher response values than mismatch probes at 100 aM (*P* < 0.001), confirming stringent low-concentration selectivity (Fig 5C). Cross-reactivity testing revealed no interference from four clinically relevant parasites (*pig tapeworm*, *Ascaris lumbricoides*, and *Clonorchis sinensis*) or eight vesicular echinococcosis-associated miRNAs (Fig 5D). A synthetic standard (10-fold serially diluted, 1 aM–100 pM) was used to generate a calibration curve (Fig 5E). The optimized one-pot system achieved specific target amplification at 1 aM within 50 minutes (Fig 5F). Real-time fluorescence monitoring (Fig 5G) yielded a detection limit of 1.41 aM and linearity across 1 aM–100 pM (Fig 5H). Visually detectable signals were observed at concentrations of≥10 aM under UV and blue light (Fig 5I), correlating strongly with isothermal amplifier results (*r* = 0.992).

### Clinical validation of cfDNA for echinococcosis

#### Clinical sample validation.

**Fig 6:**
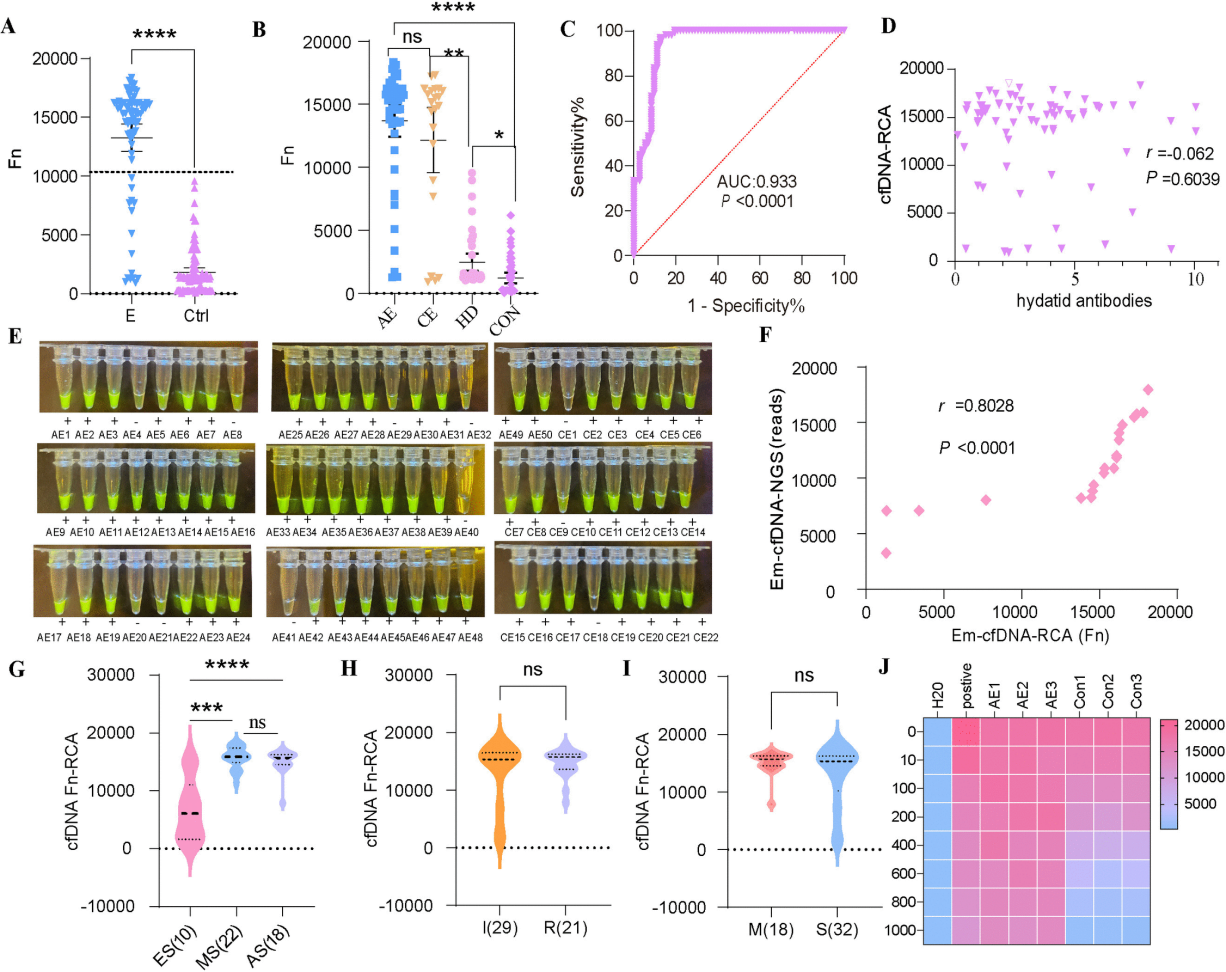
Clinical application and performance evaluation of the cfDNA-RCA method for echinococcosis diagnosis and disease monitoring. **(A):**Significant differences in cfDNA values measured by the RCA method were observed between echinococcosis patients and controls; **(B):**Subtype specificity analysis of infections; **(C):**ROC curves demonstrating diagnostic efficacy; **(D):**No significant correlation was identified between cfDNA-RCA signals and anti-*E. granulosus* antibody concentrations; **(E):**Fluorescence signals of Em-cfDNA under blue light in 50 AE and 22 cystic echinococcosis (CE) samples; **(F):**Strong positive correlation between Em-cfDNA quantified by next-generation sequencing and RCA-based detection; **(G):**Stage-dependent variations in cfDNA-Fn values (RCA method) for AE patients: late-stage patients showed significantly higher levels than early- and middle-stage patients (****P < 0.0001); **(H):**Elevated cfDNA-Fn-RCA values in recurrent infections ® compared to initial infections **(I)**; **(I):**Increased cfDNA-Fn-RCA values in metastatic AE (M) versus non-metastatic cases **(S)**.**(J):**Heatmap of Em-cfDNA fluorescence signals (RCA method) at gradient dilutions for three AE and three control samples, with color intensity reflecting RCA signal strength.AE: Alveolar echinococcosis; CE: Cystic echinococcosis; ES: Early stage; MS: Middle stage; AS: Advanced stage; M: Metastasis; NM: Non-metastatic; I: Initial infection; R: Recurrence; Ctrl: Control; ****P < 0.0001; ns: Not significant.

Fig 6A shows that patients with echinococcosis had significantly higher cfDNA values than control patients (*P* < 0.0001). Fig 6B demonstrates the difference, indicating that cfDNA values are considerably higher in echinococcosis patients and may serve as a potential biomarker for the disease, particularly in the infected subtypes. Patients with AE or CE had significantly higher cfDNA levels than the other liver disease groups and healthy controls (*P* < 0.0001). No significant difference in cfDNA levels was observed between the AE and CE patient groups.The diagnostic efficacy of cfDNA values was assessed using a ROC curve in Fig 6C, with an AUC value of 0.933 (*P* < 0.0001), suggesting that cfDNA values have high sensitivity and specificity in the diagnosis of echinococcosis. Fig 6D shows that there was no significant correlation between cfDNA values and anti-Echinococcosis antibody concentration (*r* = -0.062, *P* = 0.6039), suggesting that elevated cfDNA values may be independent of antibody levels. Fig 6E demonstrates the *Em*-cfDNA fluorescence of 50 samples from the AE and 22 samples from the CE groups under blue light. All positive samples showed significant fluorescent signals, whereas no fluorescence was observed in the negative control, suggesting that the method has reasonable specificity. Fig 6F showed a strong positive correlation (*r* = 0.8028, *P* < 0.0001) between the *Em*-cfDNA values measured by the second-generation sequencing method and the *Em*-cfDNA values detected by the RCA method, suggesting that the two methods had good concordance in detecting *Em*-cfDNA. Fig 6G shows that there was a significant difference between AE patients at different stages (early, intermediate, and late), with early-stage patients having significantly lower values than intermediate- and late-stage patients (*P* < 0.0001), suggesting that cfDNA values may be associated with disease progression. Fig 6H shows that there was no significant difference between patients with recurrence and those with initial infection, suggesting that cfDNA levels were similar in both groups. Fig 6I shows that AE patients with metastasis and those without metastasis showed no significant difference, indicating that cfDNA values were not significantly different between metastatic and non-metastatic patients. Fig 6J demonstrates the heatmap of *Em*-cfDNA fluorescence values of 3 AE and three control samples measured by the RCA method at different gradient dilutions. The color shades indicate the RCA values, and the results showed that the AE patients exhibited higher fluorescence values at various dilutions.

In comparison, the controls exhibited lower values, which further validated the high sensitivity and specificity of the RCA method.Elevated cfDNA in echinococcosis patients indicated high diagnostic efficacy and disease progression correlation, with no significant link to antibody levels or metastasis. RCA and second-generation sequencing methods agreed well on Em-cfDNA detection, both showing high sensitivity and specificity.

## Discussion

This study establishes a novel, highly sensitive CRISPR/Cas12a-RCA assay for the pan-*Echinococcus* detection of circulating cfDNA. By strategically selecting a conserved 28S rDNA fragment as a pan-*Echinococcus* biomarker, we developed a one-pot assay that achieves rapid, ultrasensitive, and equipment-free detection. Clinical validation demonstrated high accuracy in distinguishing infected individuals from controls. Crucially, the assay’s comparable detection of AE and CE—stemming directly from the conserved nature of the biomarker—is not a limitation but a design feature that prioritizes screening sensitivity over species specificity, thereby solidifying its role as a practical primary screening tool.

The high performance of our assay is underpinned by the distinctive advantages of the integrated CRISPR/Cas12a-RCA platform, which directly address the need for POCT.RCA has been predominantly applied to the detection of miRNAs, while its applications in cfDNA detection remain limited [25,28,34]. This system utilizes a CRISPR-responsive padlock probe and an optimized three-enzyme reaction network, seamlessly integrating RCA-based exponential amplification with Cas12a trans-cleavage activity into a one-tube, one-step reaction. The system’s detection limit is as low as 1 aM, surpassing the sensitivity of traditional PCR (pM level) and common isothermal techniques like LAMP (nM level) [35,36]. Visual readouts under UV/blue light (thresholds: 10 aM and 1 aM, respectively) further confirm its ultra-sensitivity [18,34]. The reaction is performed at 37°C within 50 minutes, eliminating the need for thermal cyclers required in conventional PCR. Amplification and detection are completed in a single tube, enhancing efficiency for rapid diagnostics.Operationally, the system only requires a basic thermostat and a portable UV/blue light source, avoiding reliance on costly fluorescence quantifiers, making it highly suitable for primary care or on-site use screening [18,27]. The system’s versatility is enhanced by compatibility with CRISPR/Cas and multi-probe biosensors. CRISPR’s sequence-specific cleavage minimizes non-specific amplification [37], while RCA’s high amplification efficiency compensates for CRISPR’s sensitivity limitations [38], synergistically achieving high sensitivity and specificity.

Our strategic selection of the 28S rDNA fragment and the innovative one-pot detection method overcome key limitations of previous approaches.Most current studies focus on mitochondrial genes (e.g., *cox1, nad1, nad5*) and nuclear genes (e.g., *U1 snRNA*, *ITS1/ITS2 rDNA*) [14,39–43]. While some success has been achieved with NGS-based targeted sequencing of ITS regions (sensitivity up to 80%) [43], conventional qPCR detection of targets like U1 snRNA and nad5 has shown low sensitivity (e.g., 9.7%) [42]. This bottleneck exists because highly specific longer fragments, such as the homologous Em-28S, fall outside the optimal amplification range of traditional PCR (<200 bp) [39,44]. In contrast, the conserved 34-bp region we identified within the 28S rDNA exhibits low homology with the human genome, ensuring specificity. The study developed a novel one-pot system. Unlike conventional tandem reaction strategies that risk reactant degradation [45], our integrated CRISPR/Cas12a-RCA assay avoids this pitfall, representing a significant methodological advancement.

To effectively target this sequence, we engineered a novel one-step, one-pot isothermal assay that synergistically combines RCA with the CRISPR/Cas12a system to overcome key limitations of existing methods [23,25,46]. While conventional CRISPR-based detection often relies on tandem reactions that risk reactant degradation and inhibit exponential amplification [47], our innovative approach utilizes CRISPR-responsive padlock probes [42], and an optimized three-enzyme reaction network [48]to seamlessly integrate amplification and detection. This innovative strategy not only enhances specificity by minimizing non-specific amplification [31] but also delivers exceptional, femtomolar-level sensitivity required for detecting trace cfDNA in early disease stages [24,49,50]. Furthermore, the one-pot design ensures contamination-proof operation and high cost-effectiveness (approximately $0.60 per test) [31,51], while the minimal need for sophisticated instrumentation makes this rapid, precise method particularly ideal as a convenient bedside diagnostic tool for use in remote areas [25], representing a substantial advancement in practical cfDNA assay performance.This innovative approach not only enhances the detection capabilities but also addresses the practical challenges of disease diagnosis.Detecting circulating cfDNA signals in biological samples during the early stages of disease requires high sensitivity [24]. The CRISPR/Cas12a-RCA method delivers femtomolar-level sensitivity and single-nucleotide specificity, significantly streamlining and accelerating the analysis process [49,50]. This method offers contamination-proof operation through its one-pot design and is highly cost-effective, with material costs as low as $0.60 per assay in research settings [31,51]. These features represent a substantial advancement in cfDNA assay performance. Moreover, as a convenient bedside diagnostic tool that does not require sophisticated instrumentation, this method is particularly ideal for use in remote areas [25].

This study has limitations that point to valuable future research directions. First, although clinically validated, larger multi-center cohorts will strengthen the evidence. Second, transitioning from semi-quantitative RPM to an absolute quantitative standard (e.g., via digital RCA) is a goal. Practical challenges in resource-limited settings, such as plasma volume (4 mL) and specialized collection tubes, need addressing through device miniaturization and alternative preservation methods.A positive result would trigger definitive imaging (e.g., ultrasound) for differentiation, aligning with standard clinical pathways. Finally, factors like sample processing, extraction efficiency, and patient immune status may influence cfDNA detection rates and warrant further investigation.

To address these points and advance clinical translation, future work will focus on: 1) Implementing absolute quantification (e.g., digital RCA) and miniaturizing devices to reduce sample volume; 2) Expanding validation across diverse populations and settings; 3) Exploring the utility of EM-28S cfDNA’s short half-life for postoperative monitoring; 4) Developing a multi-parasite panel for species differentiation; 5) Investigating alternative samples (e.g., saliva/urine) and the biology of parasite-derived cfDNA. Concurrently, cost-benefit analyses will be crucial for assessing scalability in resource-limited areas.

## Conclusions

The study developed a one-step isothermal CRISPR/Cas12a-RCA system for detecting *echinococcosis* parasite cfDNA in plasma. Targeting a 34 bp 28S fragment, it achieved a 1.41 aM detection limit, 87.5% sensitivity, and 96.9% specificity in clinical tests, offering a new tool for *echinococcosis* screening that is highly suitable for POCT.

## Supporting information

S1 Table Echinococcus multilocularis gene sequence information sheet. (DOCX)

S2 Table AE2 -28S Gene Sequence Alignment Results. (DOCX)

S3 Table *Em-28S* common sequence of bases per sample. (DOCX)

S4 Table RCA-CRISPR padlock 5p probe Sequences. (DOCX)

S5 Table Laboratory indicators for echinococcus and controls. (DOCX)

S1 Fig A basic flowchart of plasma cfDNA sequencing data, which focuses on the acquisition of Echinococcus sequences through the construction of the Echinococcus reference database. (DOCX)

S2 Fig An exhaustive flowchart of the microarray sequencing (chip seq) analysis of *Em*-cfDNA, which describes in detail the entire process from reference genome mapping to the analysis of the enrichment of relevant genes. (DOCX)

S3 Fig Fluorescence Signal Intensity Variations of Different Gene Sequences During RCA Amplification. (DOCX)

S4 Fig Exploration of the principle of secondary triggering of RCA products;A: schematic diagram of secondary triggering of RCA products. Shows the amplification principle of RCA products and multi-copy versus single-copy. Illustrates how paired and unpaired RCA products at the 3’ end affect the initiation of the T4-PHI29-CAS12A reaction. B: Fluorescence curves of different concentrations of Singlecopies as amplification materials.C: Fluorescence profiles of two types of degradation products used in the experiment, which were each used as amplification feedstock after in-gel recovery;D: Amplification curves of synthetic short products.
(DOCX)

S1 File Chromatin Immunoprecipitation DNA Sequencing. (DOCX)

S2 File One-Pot RCA-CRISPR Assay Protocol. (DOCX)

S3 File Optimised step-by-step RCA-CRISPR Protocol. (DOCX)
