# Supplementary material for: CRISPR/Cas12a-RCA enables ultrasensitive detection of circulating free DNA for noninvasive diagnosis of echinococcosis
Source: PLoS Negl Trop Dis. 2026 Jan 8;20(1):e0013069. doi: 10.1371/journal.pntd.0013069 (PMC12810898; doi:10.1371/journal.pntd.0013069)
Supplement: S1 Table — (DOCX) [file pntd.0013069.s001.docx]

**S1 Table. *Echinococcus multilocularis* gene sequence information sheet**

|  | **Description** | **Sequence ID** | **Length** |
| --- | --- | --- | --- |
| ***28S*** | *Echinococcus multilocularis* *28S* large subunit ribosomal RNA gene, partial sequence | AY615426.1 | 604 |
| ***18S*** | *Echinococcus multilocularis* gene for *18S rRNA*, partial sequence | [AB731634.1](https://www.ncbi.nlm.nih.gov/nucleotide/AB731634.1?report=genbank&log$=nucltop&blast_rank=1&RID=K9T1MYT0013) | 2250 |
| ***MF-2*** | [*Echinococcus multilocularis* isolate *MF-2 18S* ribosomal RNA gene, partial sequence](https://blast.ncbi.nlm.nih.gov/Blast.cgi#alnHdr_189171180) | [EU704117.1](https://www.ncbi.nlm.nih.gov/nucleotide/EU704117.1?report=genbank&log$=nucltop&blast_rank=2&RID=K9T1MYT0013) | 362 |
| ***MF-1*** | [*Echinococcus multilocularis* isolate *MF-1 18S* ribosomal RNA gene, partial](https://blast.ncbi.nlm.nih.gov/Blast.cgi#alnHdr_189171179) | EU704116.1 | 360 |
| ***YL 18S*** | [*Echinococcus multilocularis* isolate *YL 18S* ribosomal RNA gene, partial sequence](https://blast.ncbi.nlm.nih.gov/Blast.cgi#alnHdr_189171181) | [EU704118.1](https://www.ncbi.nlm.nih.gov/nucleotide/EU704118.1?report=genbank&log$=nucltop&blast_rank=4&RID=K9T1MYT0013) | 339 |
| ***emns1*** | *Echinococcus multilocularis* partial mRNA for non capsid protein 1 | [LR029140.1](https://www.ncbi.nlm.nih.gov/nucleotide/LR029140.1?report=genbank&log$=nuclalign&blast_rank=1&RID=K9T1MYT0013) | 1103 |
| ***spliced*** | *Echinococcus multilocularis* spliced leader sequence and spliced leader exon | AJ292365.1 | 1512 |
| ***species*** | *Echinococcus multilocularis* species-specific diagnostic DNA probe | M38199.1 | 609 |
| ***EmCA90*** | *Echinococcus multilocularis* clone *EmCA90* microsatellite sequence | AF492848.1 | 734 |
| ***eif4A*** | *Echinococcus multilocularis* mRNA for translation initiation factor 4A-like protein (*eif4A* gene) | AJ292369.1 | 1336 |
| ***U1*** | [E.multilocularis *U1* small nuclear RNA gene](https://blast.ncbi.nlm.nih.gov/Blast.cgi#alnHdr_158860) | [M73768.1](https://www.ncbi.nlm.nih.gov/nucleotide/M73768.1?report=genbank&log$=nuclalign&blast_rank=1&RID=K89A5BYJ013) | 1303 |
| ***FABP2*** | Echinococcus multilocularis *FABP2* mRNA, complete cds | [MN809109.1](https://www.ncbi.nlm.nih.gov/nucleotide/MN809109.1?report=genbank&log$=nuclalign&blast_rank=1&RID=K8G3GHTM013) | 402 |

**Notes:Sequence IDs**：unique identifiers in the GenBank database that are used to retrieve and reference specific gene sequences;**Length:**he number of base pairs per sequence, reflecting the relative size of the sequence.
