# Supplementary material for: CRISPR/Cas12a-RCA enables ultrasensitive detection of circulating free DNA for noninvasive diagnosis of echinococcosis
Source: PLoS Negl Trop Dis. 2026 Jan 8;20(1):e0013069. doi: 10.1371/journal.pntd.0013069 (PMC12810898; doi:10.1371/journal.pntd.0013069)
Supplement: S2 Table — (DOCX) [file pntd.0013069.s002.docx]

**S2 Table. AE2 -28S Gene Sequence Alignment Results**

| **ID** | **Query ID** | **Query Length** | **Range 1** | **Expect** | **Identities** |
| --- | --- | --- | --- | --- | --- |
| **1** | [LN901678.1:77-425](https://blast.ncbi.nlm.nih.gov/Blast.cgi) | 348bp | 1-283 | 1E-149 | 283/283(100%) |
| **2** | [LN901842.1:23700-24053](https://blast.ncbi.nlm.nih.gov/Blast.cgi) | 353bp | 1-287 | 9E-152 | 287/287(100%) |
| **3** | [LN901879.1:4111-4622](https://blast.ncbi.nlm.nih.gov/Blast.cgi) | 511bp | 559-604 | 1E-17 | 46/46(100%) |
| **4** | [LN901879.1:4896-5251](https://blast.ncbi.nlm.nih.gov/Blast.cgi) | 355bp | 1-284 | 4E-150 | 284/284(100%) |
| **5** | [LN902015.1:12097-12447](https://blast.ncbi.nlm.nih.gov/Blast.cgi) | 350bp | 1-285 | 1E-150 | 285/285(100%) |
| **6** | [LN902015.1:12738-13227](https://blast.ncbi.nlm.nih.gov/Blast.cgi) | 489bp | 577-604 | 0.0000001 | 28/28(100%) |
| **7** | [LN902162.1:5915-6264](https://blast.ncbi.nlm.nih.gov/Blast.cgi) | 349bp | 1-281 | 2E-148 | 281/281(100%) |
| **8** | [LN902166.1:874-1382](https://blast.ncbi.nlm.nih.gov/Blast.cgi) | 508bp | 558-604 | 3E-18 | 47/47(100%) |
| **9** | [LN902166.1:1650-2007](https://blast.ncbi.nlm.nih.gov/Blast.cgi) | 357bp | 1-289 | 7E-153 | 289/289(100%) |
| **10** | [LN902220.1:1655-1907](https://blast.ncbi.nlm.nih.gov/Blast.cgi) | 252bp | 1-250 | 1E-129 | 249/250(99%) |
| **11** | [LN902231.1:5429-6012](https://blast.ncbi.nlm.nih.gov/Blast.cgi) | 583bp | 561-604 | 2E-16 | 44/44(100%) |
| **12** | [LN902231.1:6284-6623](https://blast.ncbi.nlm.nih.gov/Blast.cgi) | 339bp | 1-282 | 5E-149 | 282/282(100%) |
| **13** | [LN902246.1:2035-2384](https://blast.ncbi.nlm.nih.gov/Blast.cgi) | 349bp | 1-282 | 5E-149 | 282/282(100%) |
| **14** | [LN902492.1:15248-15584](https://blast.ncbi.nlm.nih.gov/Blast.cgi) | 336bp | 1-287 | 8E-152 | 287/287(100%) |
| **15** | [LN902562.1:439-717](https://blast.ncbi.nlm.nih.gov/Blast.cgi) | 278bp | 1-221 | 3E-115 | 221/221(100%) |
| **16** | [LN902701.1:0-254](https://blast.ncbi.nlm.nih.gov/Blast.cgi) | 254bp | 1-197 | 3E-100 | 196/197(99%) |
| **17** | [LN902746.1:2626-3123](https://blast.ncbi.nlm.nih.gov/Blast.cgi) | 497bp | 567-604 | 3E-13 | 38/38(100%) |
| **18** | [LN902746.1:2626-3123](https://blast.ncbi.nlm.nih.gov/Blast.cgi) | 497bp | 1-273 | 5E-144 | 273/273(100%) |
| **19** | [LN902784.1:5880-6386](https://blast.ncbi.nlm.nih.gov/Blast.cgi) | 506bp | 560-604 | 4E-17 | 45/45(100%) |
| **20** | [LN902844.1:8089963-8090277](https://blast.ncbi.nlm.nih.gov/Blast.cgi) | 314bp | 1-295 | 1E-154 | 294/295(99%) |
| **21** | [LN902844.1:9902750-9903014](https://blast.ncbi.nlm.nih.gov/Blast.cgi) | 264bp | 21-284 | 2E-127 | 257/264(97%) |
| **22** | [LN902846.1:763169-763371](https://blast.ncbi.nlm.nih.gov/Blast.cgi) | 202bp | 82-283 | 9E-105 | 202/202(100%) |
| **23** | [LN902847.1:5202544-5203038](https://blast.ncbi.nlm.nih.gov/Blast.cgi) | 494bp | 577-604 | 0.0000001 | 28/28(100%) |
| **24** | [LN902847.1:5203321-5203516](https://blast.ncbi.nlm.nih.gov/Blast.cgi) | 195bp | 93-285 | 2E-96 | 191/193(99%) |
| **25** | [LN902847.1:5293286-5293488](https://blast.ncbi.nlm.nih.gov/Blast.cgi) | 202bp | 90-285 | 4E-98 | 194/196(99%) |
| **26** | [LN902847.1:5293767-5294263](https://blast.ncbi.nlm.nih.gov/Blast.cgi) | 496bp | 577-604 | 0.0000001 | 28/28(100%) |
| **27** | [LN902848.1:6038755-6039113](https://blast.ncbi.nlm.nih.gov/Blast.cgi) | 358bp | 1-287 | 9E-152 | 287/287(100%) |
| **28** | [LN902848.1:6147661-6148020](https://blast.ncbi.nlm.nih.gov/Blast.cgi) | 359bp | 1-288 | 3E-152 | 288/288(100%) |
| **common sequence** | **93-197** | **104bp** | **577-604** | **27bp** |  |

| **Notes: E-value**： the probability of getting a matching result at least as good as or better than the current matching result in  a random situation. the smaller the E-value is, the more reliable the matching result is;  **Identity：**Sequence similarity (Identity) indicates the degree of match between the query sequence and the sequence in the database, usually expressed as a percentage. 100% means a perfect match. |
| --- |
