# Supplementary material for: CRISPR/Cas12a-RCA enables ultrasensitive detection of circulating free DNA for noninvasive diagnosis of echinococcosis
Source: PLoS Negl Trop Dis. 2026 Jan 8;20(1):e0013069. doi: 10.1371/journal.pntd.0013069 (PMC12810898; doi:10.1371/journal.pntd.0013069)
Supplement: S3 Table — (DOCX) [file pntd.0013069.s003.docx]

**S3 Table. 28S common sequence of bases per sample**

| **Sample ID** | **28S-numbers** | **28S-Rang 1** | **Length** | **28S-Rang 2** | **Length** |
| --- | --- | --- | --- | --- | --- |
| **AE2** | 28 | 82-197 | 115bp | 570-604 | 34bp |
| **AE4** | 25 | 131-218 | 87bp | 568-604 | 36bp |
| **AE5** | 21 | 122-197 | 75bp | 577-604 | 27bp |
| **AE6** | 20 | 123-266 | 143bp | 577-604 | 27bp |
| **AE7** | 22 | 123-266 | 143bp | 577-604 | 27bp |
| **AE8** | 26 | 93-208 | 115bp | 569 -604 | 27bp |
| **AE9** | 29 | 108-178 | 70bp | 577-604 | 27bp |
| **AE10** | 33 | 115-186 | 71bp | 576-604 | 28bp |
| **AE11** | 32 | 106-173 | 67bp | 570-604 | 34bp |
| **AE12** | 34 | 113-186 | 73bp | 577 -604 | 27bp |
| **AE13** | 23 | 86-169 | 83bp | 577 -604 | 27bp |
| **AE14** | 22 | 71-145 | 92bp | 577 -604 | 27bp |
| **AE15** | 30 | 87-179 | 92bp | 576 -604 | 28bp |
| **AE16** | 28 | 110-179 | 69bp | 577 -604 | 27bp |
| **AE17** | 32 | 93-186 | 93bp | 576 -604 | 32bp |
| **AE18** | 31 | 97-185 | 88bp | 576 -604 | 28bp |
| **AE19** | 29 | 136-177 | 41bp | 577 -604 | 27bp |
| **AE21** | 29 | 93-197 | 104bp | 577 -604 | 27bp |
| **AE22** | 33 | 121-186 | 65bp | 568-604 | 36bp |
| **AE24** | 27 | 115-175 | 60bp | 577 -604 | 27bp |
| **common sequence** |  | **136-169** | 34bp | **577 -604** | 27bp |

**Notes:Sample ID**：the different sample；28S-numbers: the number of copies of the 28S rRNA gene in each sample.28S-Rang 1 and 28S-Rang 2:the base ranges of two different regions in the 28S rRNA gene；**length**: the length of base pairs within each specified region；common sequence: Indicates a common sequence fragment of the 28S rRNA gene that occurs in all samples.
