# Supplementary material for: CRISPR/Cas12a-RCA enables ultrasensitive detection of circulating free DNA for noninvasive diagnosis of echinococcosis
Source: PLoS Negl Trop Dis. 2026 Jan 8;20(1):e0013069. doi: 10.1371/journal.pntd.0013069 (PMC12810898; doi:10.1371/journal.pntd.0013069)
Supplement: S4 Table — (DOCX) [file pntd.0013069.s004.docx]

**S4 Table. RCA-CRISPR padlock 5p probe Sequences**

| **ID** | **RCA-CRISPR padlock 5p probe Sequences** |
| --- | --- |
| **Padlock 1** | 5p-AGGGATCTCGTGTGGAcattatatgatcgagagttgcccgcatgtgtttcGCTAGATAGTAGATAGGGACAGT |
| **Padlock 2** | 5p-TAGGGATCTCGTGTGGAcattatatgatcgagagttgcccgcatgtgtttcGCTAGATAGTAGATAGGGACAG |
| **Padlock 3** | 5p-GTAGGGATCTCGTGTGGAcattatatgatcgagagttgcccgcatgtgtttcGCTAGATAGTAGATAGGGACA |
| **Padlock 4** | 5p-AGTAGGGATCTCGTGTGGAcattatatgatcgagagttgcccgcatgtgtttcGCTAGATAGTAGATAGGGAC |
| **Padlock 5** | 5p-CAGTAGGGATCTCGTGTGGAcattatatgatcgagagttgcccgcatgtgtttcGCTAGATAGTAGATAGGGA |
| **Padlock 6** | 5p-ACAGTAGGGATCTCGTGTGGAcattatatgatcgagagttgcccgcatgtgtttcGCTAGATAGTAGATAGGG |
| **Padlock 7** | 5p-GACAGTAGGGATCTCGTGTGGAcattatatgatcgagagttgcccgcatgtgtttcGCTAGATAGTAGATAGG |
