# Supplementary material for: CRISPR/Cas12a-RCA enables ultrasensitive detection of circulating free DNA for noninvasive diagnosis of echinococcosis
Source: PLoS Negl Trop Dis. 2026 Jan 8;20(1):e0013069. doi: 10.1371/journal.pntd.0013069 (PMC12810898; doi:10.1371/journal.pntd.0013069)
Supplement: S5 Table — (DOCX) [file pntd.0013069.s005.docx]

**S5 Table.** Laboratory indicators for echinococcus and controls

| **Items** | **n** | **Echinococcus group** **(n=72)** | **n** | **Con group(n=72)** | ***t*** | ***P*** |
| --- | --- | --- | --- | --- | --- | --- |
| **AEAb** | 72 | 3.560±2.421 | 96 | 0.710±0.611 | -9.689 | <0.001 |
| **ALT** | 72 | 131.722±222.535 | 96 | 58.031±97.278 | -2.610 | 0.011 |
| **AST** | 72 | 113.694±199.120 | 96 | 48.542±35.638 | -2.725 | 0.008 |
| **ALB** | 72 | 35.636±4.772 | 96 | 35.161±4.145 | -0.684 | 0.495 |
| **ALP** | 72 | 233.115±278.864 | 96 | 166.292±169.746 | -1.909 | 0.058 |
| **cfDNA** | 72 | 13245.544±4936.920 | 96 | 2164.689±3129.412 | -16.585 | <0.001 |

**Notes:**EAb: Echinococcus antibody；ALT：Alanine Aminotransferase；AST：Aspartate Aminotransferase；ALB：Albumin；ALP：Alkaline Phosphatase；cfDNA：Cell-free DNA
