# Supplementary material for: CRISPR/Cas12a-RCA enables ultrasensitive detection of circulating free DNA for noninvasive diagnosis of echinococcosis
Source: PLoS Negl Trop Dis. 2026 Jan 8;20(1):e0013069. doi: 10.1371/journal.pntd.0013069 (PMC12810898; doi:10.1371/journal.pntd.0013069)
Supplement: S1 Fig — (DOCX) [file pntd.0013069.s006.docx]

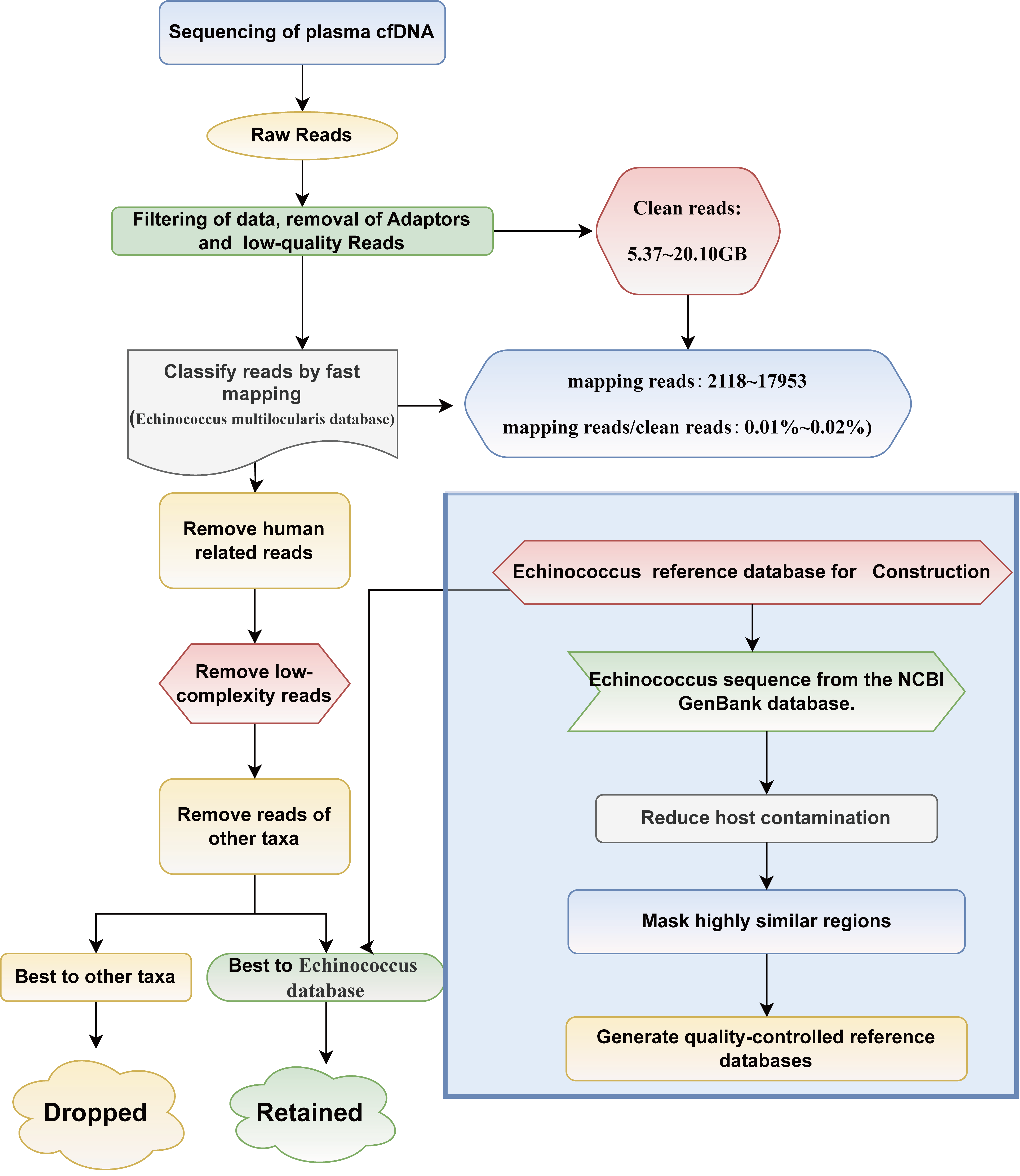


**S1 Fig.** A basic flowchart of plasma cfDNA sequencing data, which focuses on the acquisition of Echinococcus sequences through the construction of the Echinococcus reference database
