# Supplementary material for: CRISPR/Cas12a-RCA enables ultrasensitive detection of circulating free DNA for noninvasive diagnosis of echinococcosis
Source: PLoS Negl Trop Dis. 2026 Jan 8;20(1):e0013069. doi: 10.1371/journal.pntd.0013069 (PMC12810898; doi:10.1371/journal.pntd.0013069)
Supplement: S2 Fig — (DOCX) [file pntd.0013069.s007.docx]

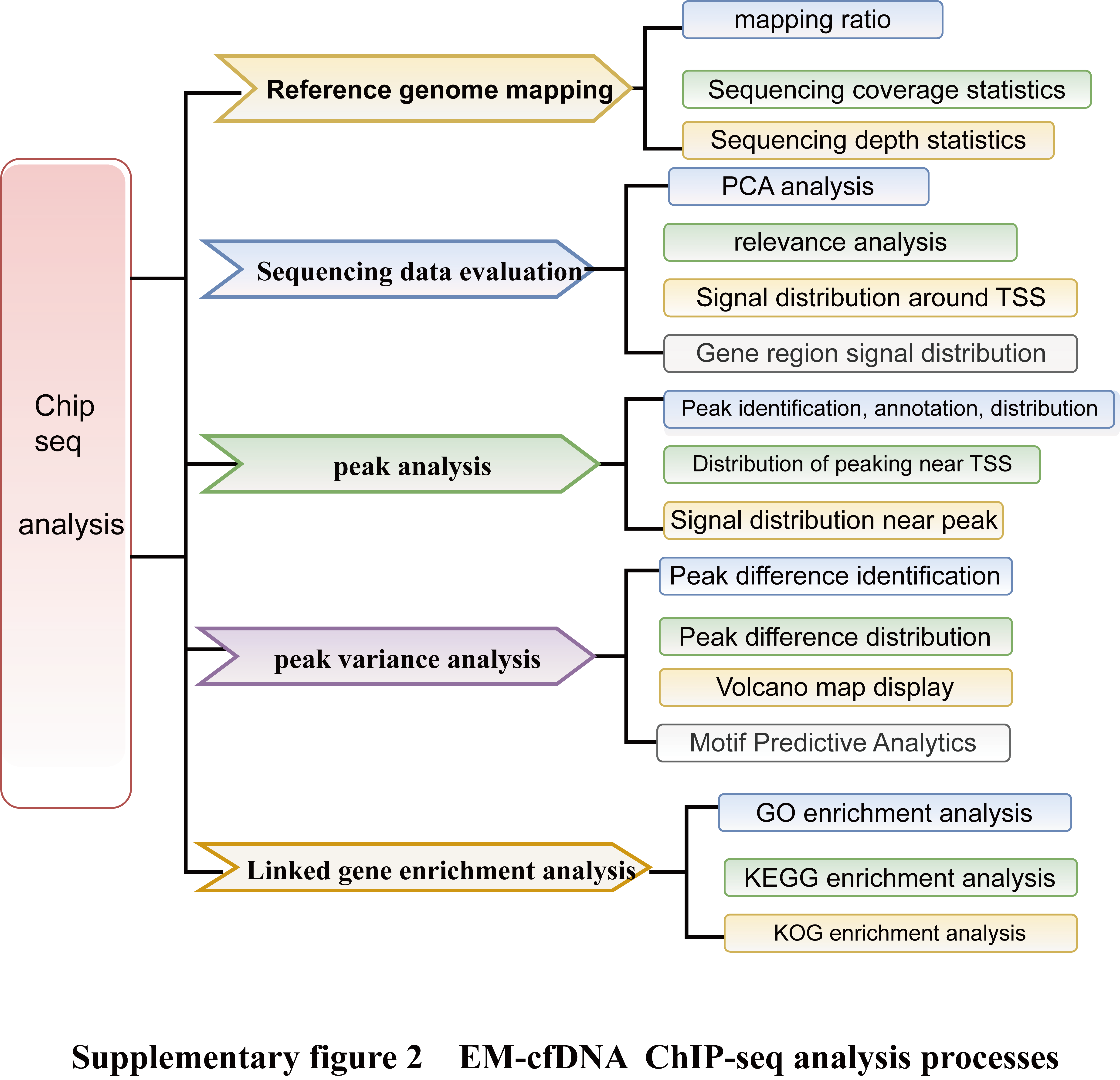


**S2 Fig.** An exhaustive flowchart of the microarray sequencing (chip seq) analysis of *Em*-cfDNA, which describes in detail the entire process from reference genome mapping to the analysis of the enrichment of relevant genes ;
