# Supplementary material for: CRISPR/Cas12a-RCA enables ultrasensitive detection of circulating free DNA for noninvasive diagnosis of echinococcosis
Source: PLoS Negl Trop Dis. 2026 Jan 8;20(1):e0013069. doi: 10.1371/journal.pntd.0013069 (PMC12810898; doi:10.1371/journal.pntd.0013069)
Supplement: S3 Fig — (DOCX) [file pntd.0013069.s008.docx]

| 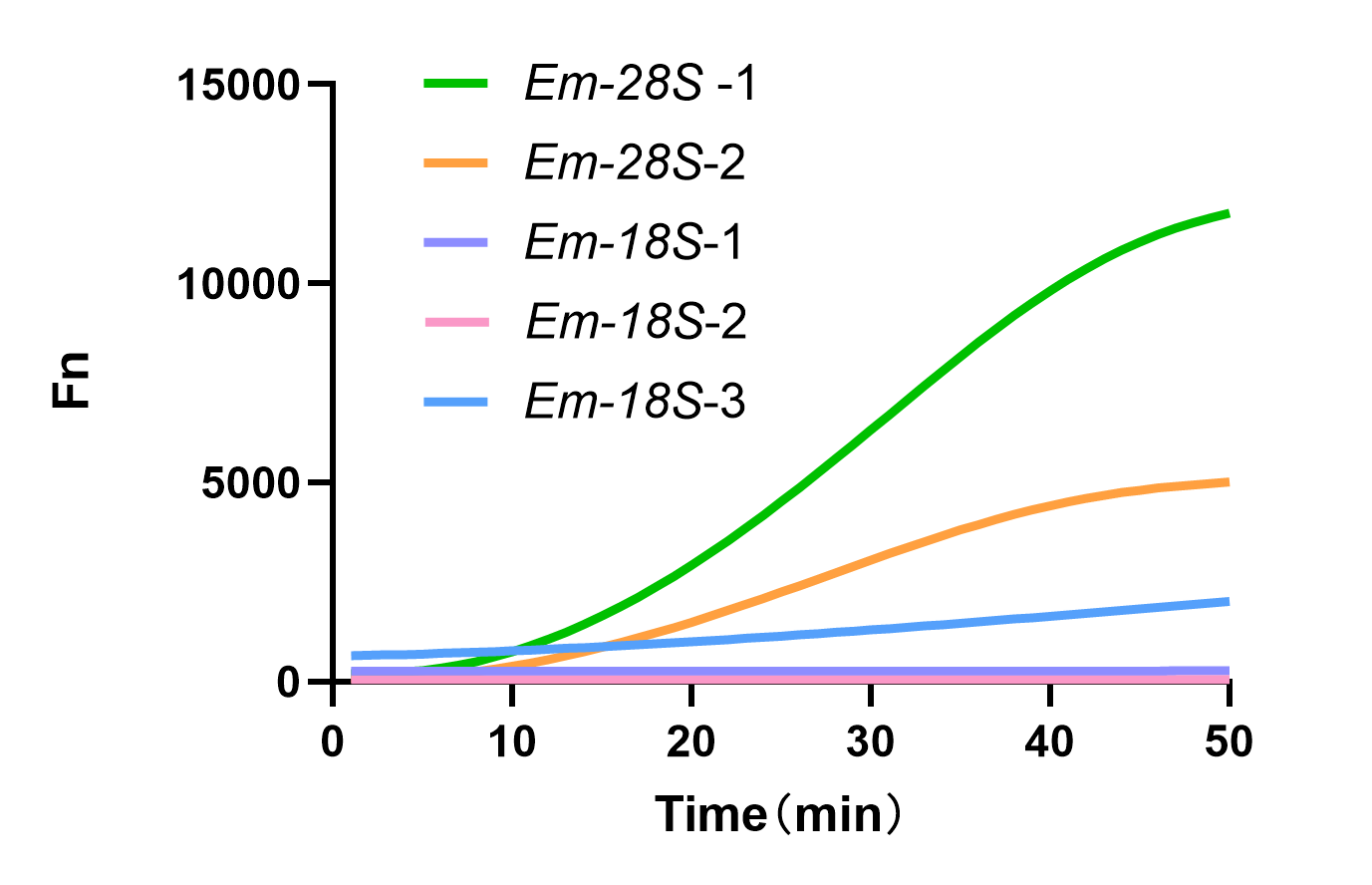 |
| --- |
| **S3 Fig**: **Fluorescence Signal Intensity Variations of Different Gene Sequences During PCR Amplification.**  This figure illustrates the changes in fluorescence signal intensity (Fn) of various gene sequences over time (minutes) during the PCR amplification process. Fluorescence signal intensity serves as an indicator of the accumulation of PCR products, reflecting the efficiency and performance of each gene sequence during amplification. By comparing the slopes and final signal intensities reached by different curves, the suitability of each gene sequence as a potential biomarker can be assessed. |
