# Supplementary material for: CRISPR/Cas12a-RCA enables ultrasensitive detection of circulating free DNA for noninvasive diagnosis of echinococcosis
Source: PLoS Negl Trop Dis. 2026 Jan 8;20(1):e0013069. doi: 10.1371/journal.pntd.0013069 (PMC12810898; doi:10.1371/journal.pntd.0013069)
Supplement: S4 Fig — Shows the amplification principle of RCA products and multi-copy versus single-copy. Illustrates how paired and unpaired RCA products at the 3’ end affect the initiation of the T4-PHI29-CAS12A reaction. B: Fluorescence curves of different concentrations of Singlecopies as amplification materials.C: Fluorescence profiles of two types of degradation products used in the experiment, which were each used as amplification feedstock after in-gel recovery;D: Amplification curves of synthetic short products. (DOCX) [file pntd.0013069.s009.docx]

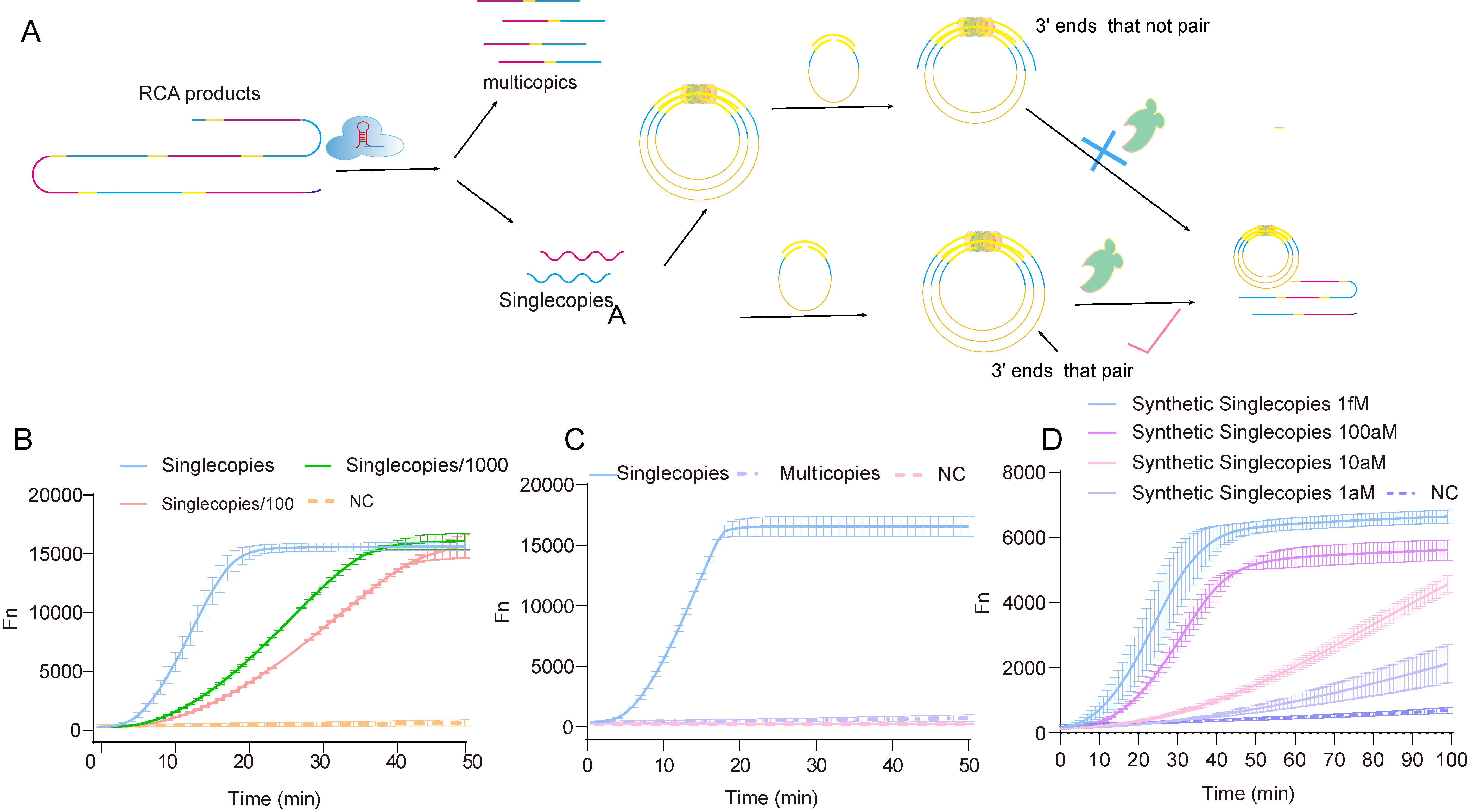


**S4 Fig.** Exploration of the principle of secondary triggering of RCA products;**A:** schematic diagram of secondary triggering of RCA products.Shows the amplification principle of RCA products and multi-copy versus single-copy. Illustrates how paired and unpaired RCA products at the 3' end affect the initiation of the T4-PHI29-CAS12A reaction. **B:** Fluorescence curves of different concentrations of Singlecopies as amplification materials.

**C:** Fluorescence profiles of two types of degradation products used in the experiment, which were each used as amplification feedstock after in-gel recovery;**D:** Amplification curves of synthetic short products.
