# Supplementary material for: CRISPR/Cas12a-RCA enables ultrasensitive detection of circulating free DNA for noninvasive diagnosis of echinococcosis
Source: PLoS Negl Trop Dis. 2026 Jan 8;20(1):e0013069. doi: 10.1371/journal.pntd.0013069 (PMC12810898; doi:10.1371/journal.pntd.0013069)
Supplement: S1 File — (DOCX) [file pntd.0013069.s010.docx]

**S1 Method.** Chromatin Immunoprecipitation DNA Sequencing.

ChIP-Seq libraries were prepared using DNA fragments enriched through antibody-mediated chromatin immunoprecipitation. To minimize false-positive signals, input DNA (non-immunoprecipitated) was sequenced in parallel as a background control.The library preparation workflow included the following steps: DNA ends were first repaired and 3’ adenine-tailed using an End Prep enzyme mix. Adapters were ligated to the fragments using ligation enhancers and T4 DNA ligase. Indexing primers were incorporated via PCR amplification, followed by size selection of ~400 bp products using magnetic beads. Library quality was verified by quantifying DNA concentration (Qubit 4.0, Thermo Scientific) and assessing fragment size distribution (2% agarose gel electrophoresis).Pooled libraries were sequenced on either a NovaSeq 6000 (Illumina) or DNBseq-T7 (BGI) platform with 2×150 bp paired-end reads, following manufacturer protocols. All ChIP-Seq experiments, including cfDNA sequencing and downstream analyses, were conducted by Bioengineering Technology Co., Ltd. (Shanghai, China).
