# Supplementary material for: CRISPR/Cas12a-RCA enables ultrasensitive detection of circulating free DNA for noninvasive diagnosis of echinococcosis
Source: PLoS Negl Trop Dis. 2026 Jan 8;20(1):e0013069. doi: 10.1371/journal.pntd.0013069 (PMC12810898; doi:10.1371/journal.pntd.0013069)
Supplement: S2 File — (DOCX) [file pntd.0013069.s011.docx]

**S2 Method.** One-Pot RCA-CRISPR Assay Protocol.

## RNP Complex Preparation:A 10 μL mixture containing 5 μL *Em* Probe 7 (100 nM) and 1 μL Phi29 buffer was prepared in a PCR tube on ice. Subsequently, 4 μL cfDNA template was added.Template Denaturation:The mixture was denatured at 85°C for 5 min, followed by rapid cooling to 25°C for probe annealing.Reaction Assembly:2 μL Phi29 buffer，2 μL BSA (10 mg/mL)，1 μL DTT (30 mM)，1 μL FAM reporter (10 μM)，1 μL HEX reporter (10 μM)，2 μL dNTPs (10 mM)，0.1 μL Phi29 DNA polymerase (10 U/μL)，0.5 μL T4 DNA ligase (1000 U/μL)，0.4 μL DEPC-treated water；Cas12a/crRNA Complex Formation：crRNA (10 μM) and Cas12a protein (10 μM) were mixed at a 1:2 molar ratio (final 0.1 μM) and incubated for 10 min at 25°C to form functional RNP complexes.Reaction Initiation5 μL of the RNP mixture was added to each reaction well. The full 20 μL system was immediately transferred to a qPCR instrument preheated to 37°C.Fluorescence signals (FAM/HEX channels) were recorded every 60 sec for 60 min to track Cas12a-mediated cleavage of RCA-amplified targets.
