# Supplementary material for: CRISPR/Cas12a-RCA enables ultrasensitive detection of circulating free DNA for noninvasive diagnosis of echinococcosis
Source: PLoS Negl Trop Dis. 2026 Jan 8;20(1):e0013069. doi: 10.1371/journal.pntd.0013069 (PMC12810898; doi:10.1371/journal.pntd.0013069)
Supplement: S3 File — (DOCX) [file pntd.0013069.s012.docx]

**S3 Method.** Optimised step-by-step RCA-CRISPR Protocol.

## The RCA-CRISPR experimental procedure is optimized through time-controlled enzymatic reactions conducted in three distinct formats:Three-step method:T4 DNA ligase is added and incubated at 37°C for 30–120 min.Phi29 DNA polymerase is introduced, followed by incubation at 37°C for 30–120 min.Cas12a enzyme is added, and the reaction is immediately analyzed. Two-step method:Protocol 1:T4 DNA ligase and phi29 DNA polymerase are combined simultaneously and incubated at 37°C for 30–120 min.Cas12a enzyme is added for subsequent analysis；Protocol 2:T4 DNA ligase is first incubated at 37°C for 30–120 min.Phi29 DNA polymerase and Cas12a enzyme are then added for simultaneous incubation and analysis. One-pot method:T4 DNA ligase, phi29 DNA polymerase, and Cas12a enzyme are mixed in a single reaction tube.The mixture is incubated at 37°C for 30–120 min before gel electrophoresis analysis.All protocols systematically evaluate the impact of reaction duration (30/60/90/120 min) and stepwise enzyme addition on RCA amplification efficiency.
